# Supplementary material for: FGF1 ameliorates hepatic steatosis through acute activation of the unfolded protein response and VLDL production
Source: JHEP Rep. 2025 Oct 30;8(2):101660. doi: 10.1016/j.jhepr.2025.101660 (PMC12800356; doi:10.1016/j.jhepr.2025.101660)
Supplement: Multimedia component 1 [file mmc1.pdf]

FGF1 ameliorates hepatic steatosis through acute activation of the unfolded protein response and VLDL production

Tim van Zutphen, Dicky Struik, Weilin Liu Sihao Liu, Benan Pelin Sermikli, Justina C. Wolters, Henkjan J. Verkade, Annette R Atkins, Michael Downes, Ronald M. Evans, Johan W. Jonker

Table of contents

Fig. S1……………………………………………………………………………………2

Fig. S2……………………………………………………………………………………3

Fig. S3……………………………………………………………………………………5

Fig. S4……………………………………………………………………………………7

Fig. S5……………………………………………………………………………………8

Fig. S6……………………………………………………………………………………9

Table S1………………………………………………………………………………..10

Table S2………………………………………………………………………………..11

Fig. S1

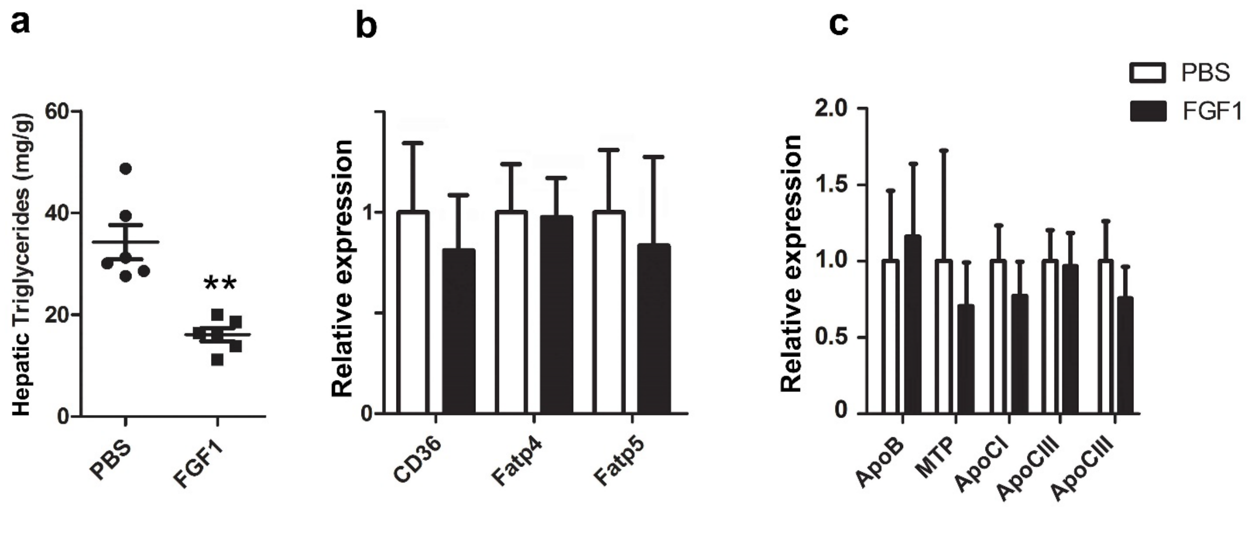

Fig. S1 (a) Hepatic triglyceride levels of *ob/ob* mice treated for 2 weeks with FGF1. (b) Hepatic expression levels of fatty acid transporter genes *Cd36*, *Fatp4* and *Fatp5* and (c) apolipoprotein genes 14-hours after FGF1 stimulation in *ob/ob*. All experiments n=6-8.

Fig. S2

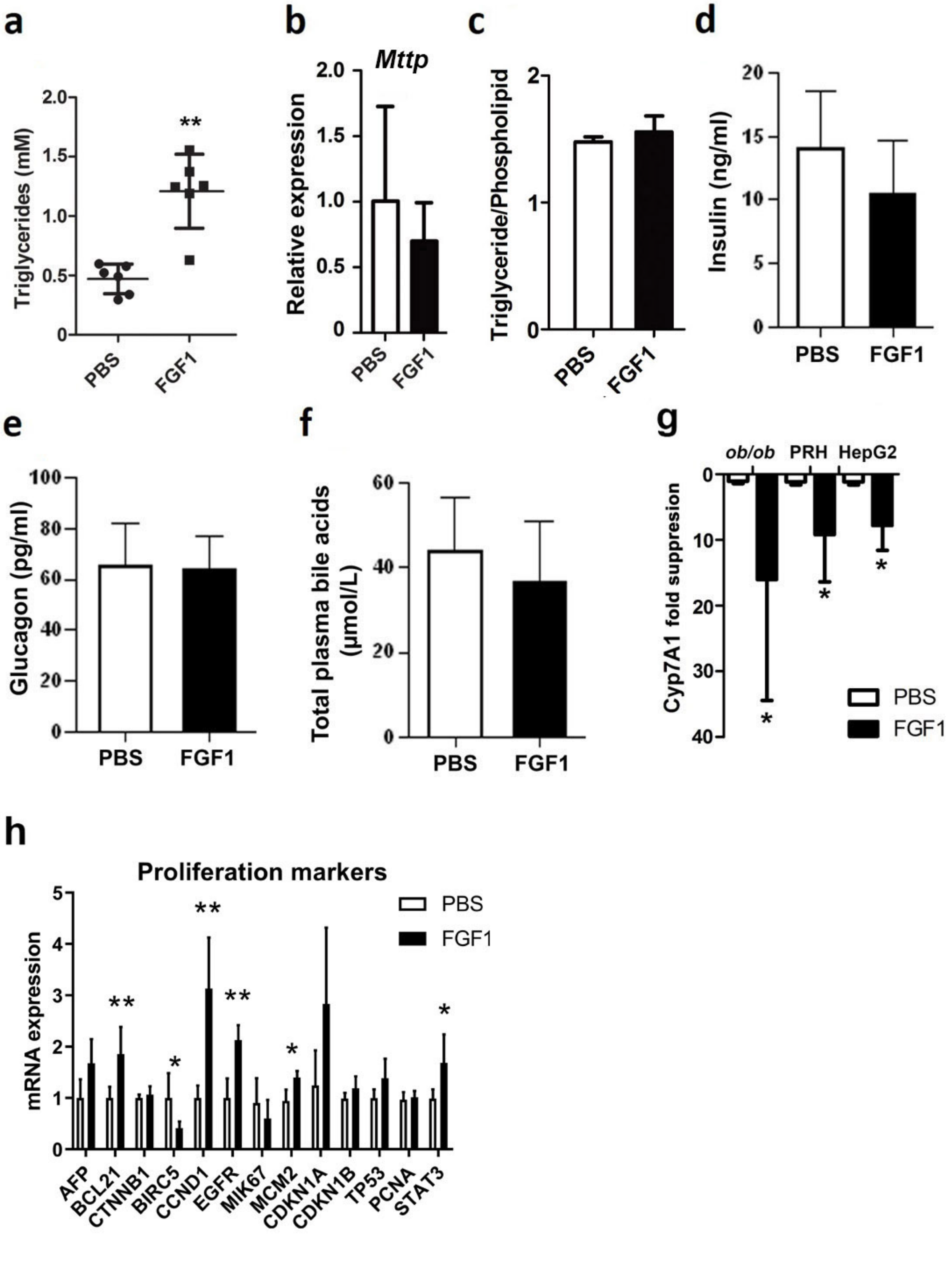

a
Triglycerides (mM)
**
PBS
FGF1
b
Mttp
Relative expression
PBS
FGF1
c
Triglyceride/Phospholipid
PBS
FGF1
d
Insulin (ng/ml)
PBS
FGF1
e
Glucagon (pg/ml)
PBS
FGF1
f
Total plasma bile acids (μmol/L)
PBS
FGF1
g
ob/ob
PRH
HepG2
Cyp7A1 fold suppresion
PBS
FGF1
h
Proliferation markers
mRNA expression
PBS
FGF1
AFP
BCL21
CTNNB1
BIRC5
CCND1
EGFR
MIK67
MCM2
CDKN1A
CDKN1B
TP53
PCNA
STAT3

Fig. S2. (a) Plasma triglycerides of *ob/ob* 24-hours after the last FGF1 administration of a 2-week treatment (Mann-Whitney U-test), **p<0,01. (b) Hepatic expression levels of microsomal triglyceride transfer protein (*Mttp*) in *ob/ob* 14 hours after an FGF1 injection. (c) Plasma insulin (d), plasma glucagon (e), and total bile acid concentrations (f) 24 hours after injection in *ob/ob.* (g) Suppression of bile acid synthesis gene *Cyp7A1* expression in *ob/ob* liver, primary rat hepatocytes and HepG2 by FGF1 (after 14, 16 & 16 hours respectively, Mann-Whitney U-test), *p<0,05. (h) Proliferation marker gene expression in *ob/ob* liver 6 hours after FGF1 injection. All experiments n=6-8.

Fig. S3

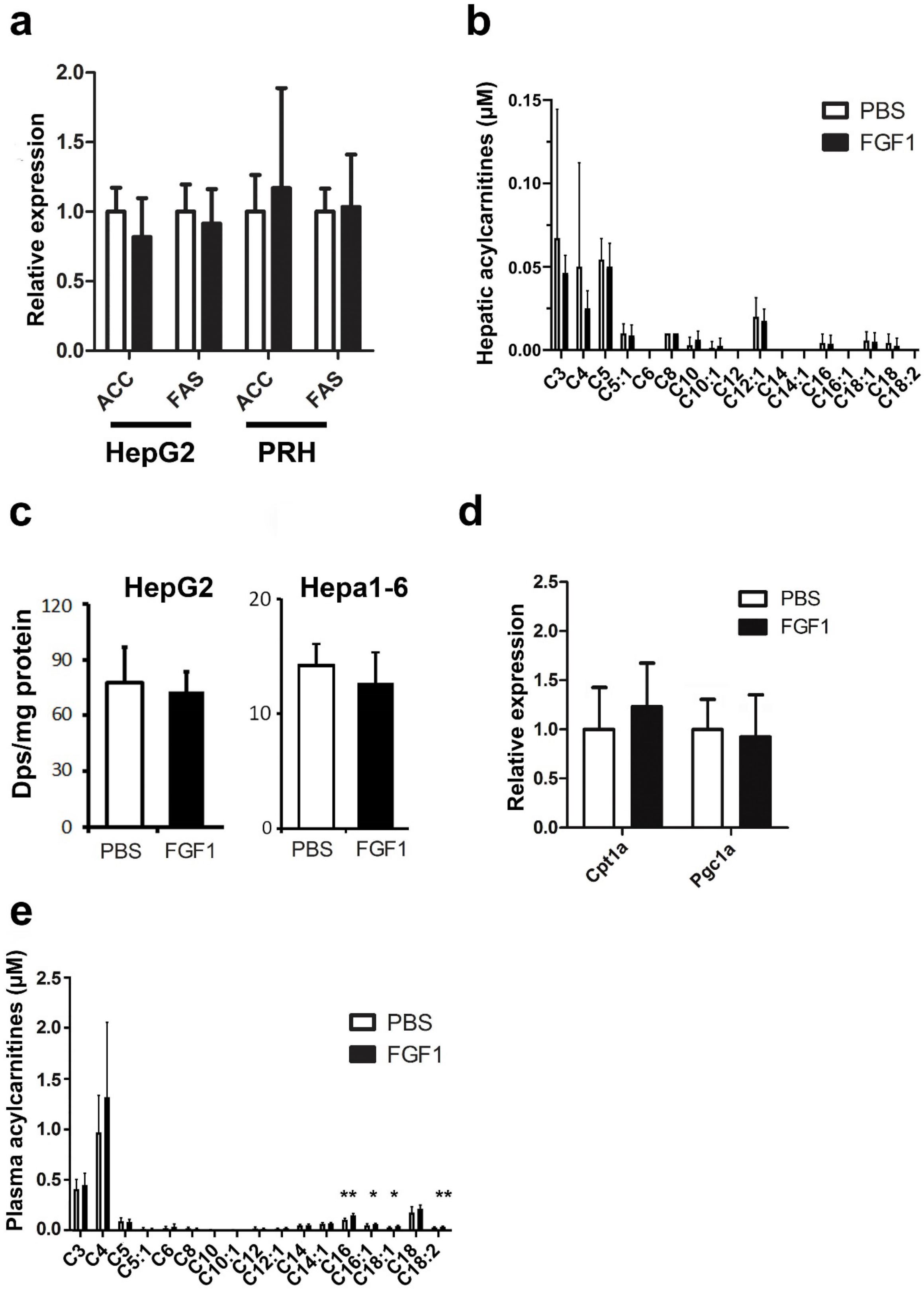
a
Relative expression
2.0
1.5
1.0
0.5
0.0
ACC
FAS
ACC
FAS
HepG2
PRH
b
Hepatic acylcarnitines (μM)
0.15
0.10
0.05
0.00
PBS
FGF1
C3
C4
C5
C5:1
C6
C8
C10
C10:1
C12
C12:1
C14
C14:1
C16
C16:1
C18:1
C18
C18:2
c
Dps/mg protein
HepG2
Hepa1-6
120
90
60
30
0
20
10
0
PBS
FGF1
PBS
FGF1
d
Relative expression
2.5
2.0
1.5
1.0
0.5
0.0
PBS
FGF1
Cpt1a
Pgc1a
e
Plasma acylcarnitines (μM)
2.5
2.0
1.5
1.0
0.5
0.0
PBS
FGF1
**
*
*
**
C3
C4
C5
C5:1
C6
C8
C10
C10:1
C12
C12:1
C14
C14:1
C16
C16:1
C18:1
C18
C18:2

Fig. S3. (a) Expression levels of lipogenesis genes Acetyl-CoA carboxylase (*Acc*) and Fatty acid synthase (*Fas*) in 6h FGF1-stimulated HepG2 cells and primary rat hepatocytes. (b) Acyl carnitines in liver of 14h FGF1-treated *ob/ob* mice. (c) β-oxidation rates in isolated mitochondria from HepG2 and Hepa1-6 cells after 6h FGF1 administration. (d) Hepatic expression levels of mitochondrial regulators *Cpt1a* and *Pgc1α* in 14h treated *ob/ob* mice. (e) Acyl carnitines in plasma of 14h FGF1-treated *ob/ob* mice (Mann-Whitney U-test), *p<0,05, **p<0,01. All experiments n=6-8.

Fig. S4

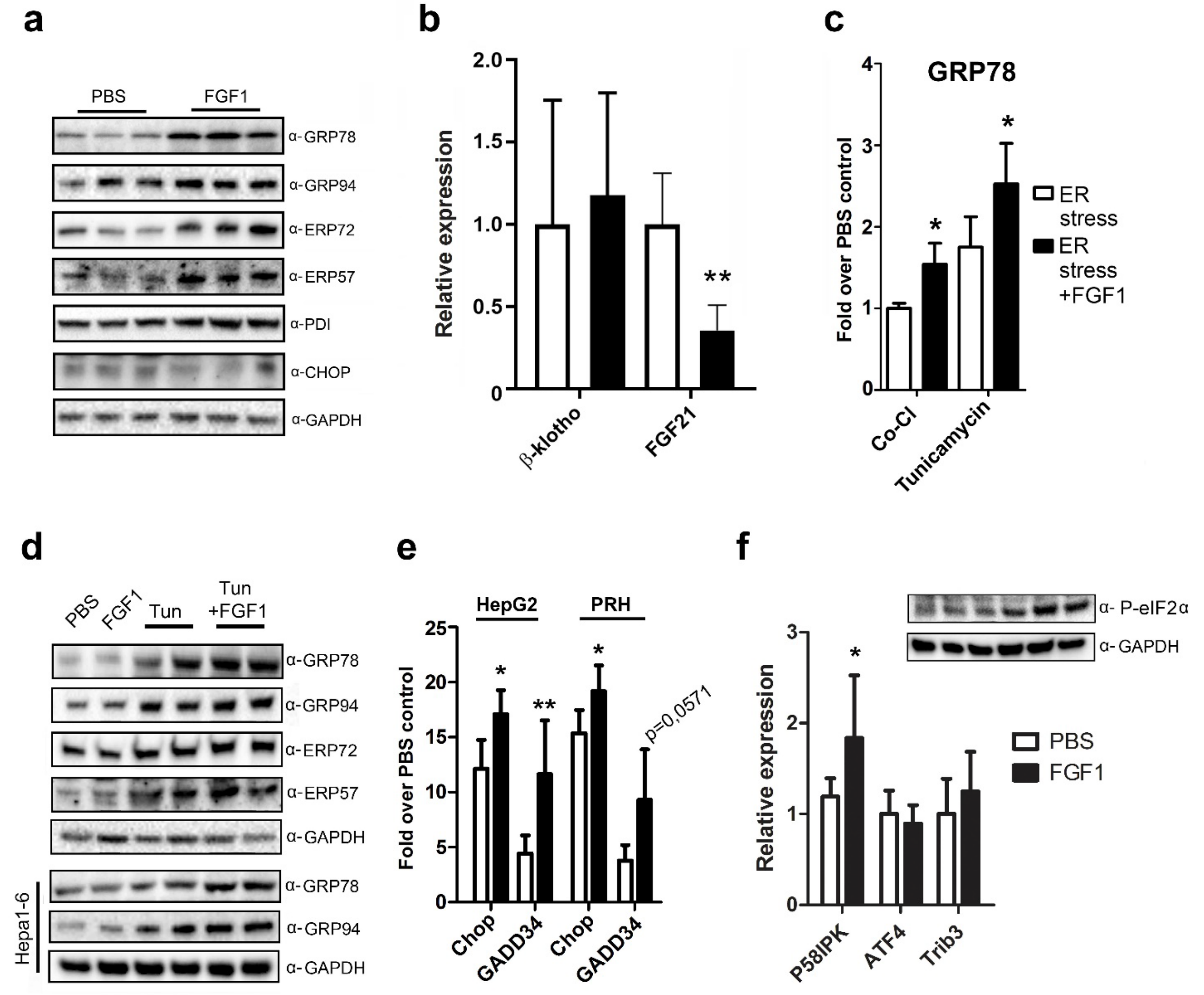

Fig. S4 (a) Hepatic UPR chaperone protein levels 24-hrs after last injection of a two-week FGF1 treatment in *ob/ob* mice. (b) Expression of *β-klotho* and *Fgf21* in same livers. (c) *Grp78 h*yperactivation in HepG2 by FGF1 upon 3-hour pretreatment with stressors such as cobalt and tunicamycin (Mann-Whitney U-test), *p<0,05. (d) Hyperactivation in HepG2 of UPR chaperone protein levels of Grp78, Grp94, Erp72 and Erp57 as well as in Hepa1-6 cells upon pretreatment with tunicamycin. (e) Hyperactivation of Perk-Atf4-Chop pathway target genes *Chop* and *Gadd34* by FGF1 in HepG2 and primary rat hepatocytes upon glucosamine pre-treatment (that is not observed *in vivo* in Figure 4A,B or Fig S4F, Mann-Whitney U-test), *p<0,05, **p<0,01. (f) Phosphorylation of eIF2α, but not induction of downstream targets *ATF4* and *Trib3* in livers of 14h FGF1-treated *ob/ob* mice (Mann-Whitney U-test), *p<0,05. All experiments n=6-8.

Fig. S5

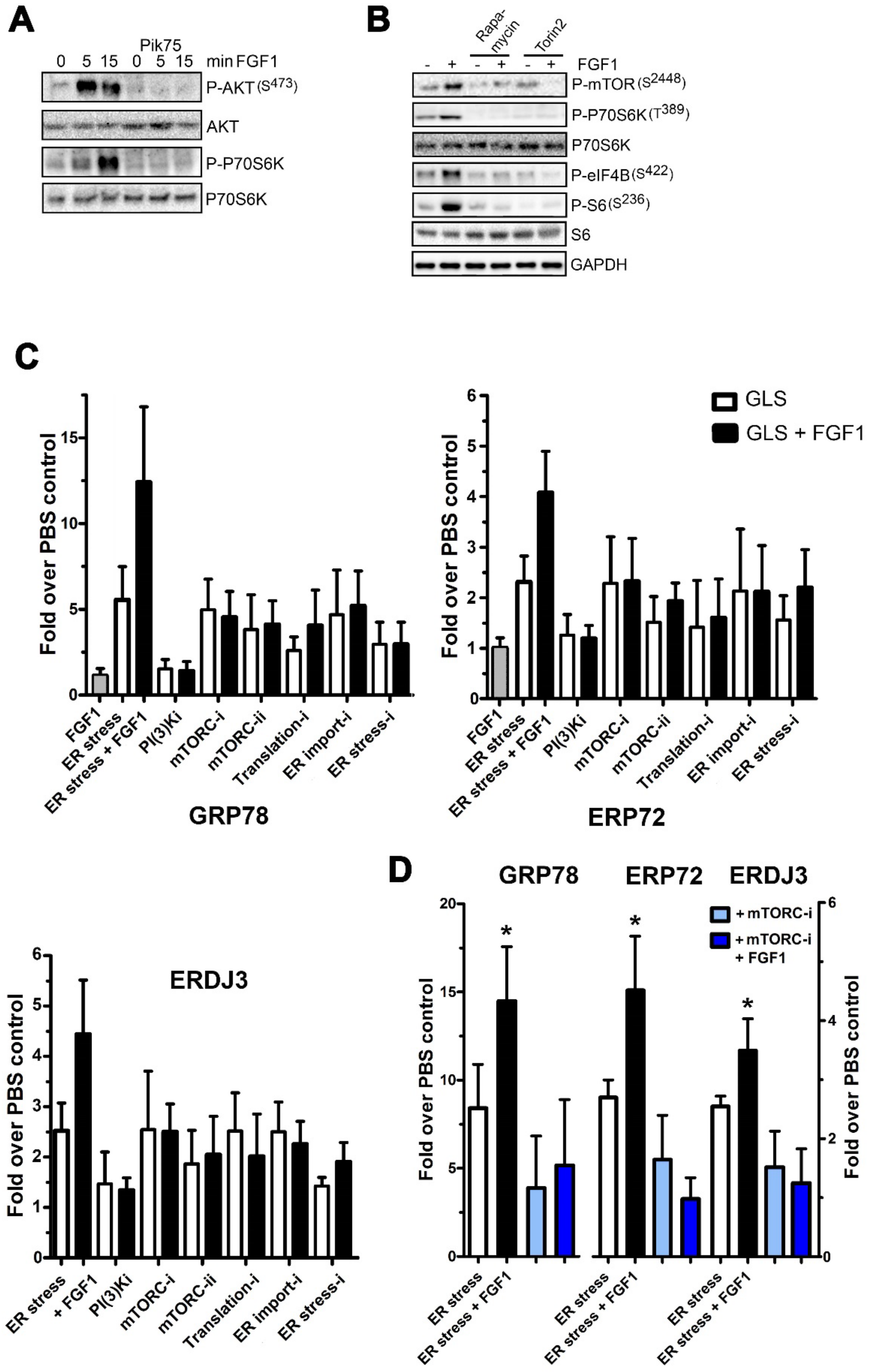
A
Pik75
0 5 15 0 5 15 min FGF1
P-AKT (S473)
AKT
P-P70S6K
P70S6K
B
Rapa-mycin
Torin2
- + - + - + FGF1
P-mTOR (S2448)
P-P70S6K (T389)
P70S6K
P-eIF4B (S422)
P-S6 (S236)
S6
GAPDH
C
GLS
GLS + FGF1
Fold over PBS control
FGF1
ER stress
ER stress + FGF1
PI(3)Ki
mTORC-i
mTORC-ii
Translation-i
ER import-i
ER stress-i
GRP78
ERP72
ERDJ3
D
GRP78
ERP72
ERDJ3
+ mTORC-i
+ mTORC-i + FGF1
ER stress
ER stress + FGF1

Fig. S5 (a) Suppressive effect of PI3K inhibition (by PIK-75) on 15 min FGF1-stimulated phosphorylation of AKT and P70S6K in HepG2 cells; (b) Suppressive effect of mTOR inhibition (by Rapamycin and Torin-2) on 15 min FGF1-stimulated phosphorylation of P70S6K, eIF4B and S6 in HepG2 cells. (c) Expanded representation of Figure 4D, suppression of hyperactivation of UPR chaperones *Grp78*, *Erp72* and *Erdj3*, including the controls that received the indicated inhibitors without the subsequent FGF1 stimulation. (d) Suppressive effect of mTOR inhibition (by Rapamycin and Torin-2) on hyperactivation of the UPR chaperones *Grp78*, *Grp94* and *Erdj3* in primary rat hepatocytes (Mann-Whitney U-test), *p<0,05, **p<0,01. All experiments n=6-12.

Fig. S6

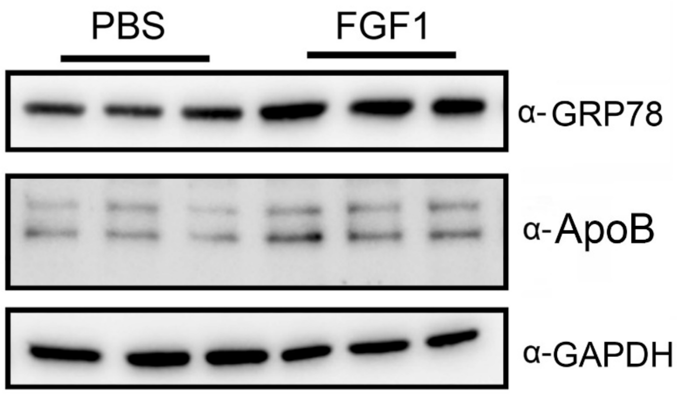

Fig. S6 Grp78 and ApoB protein levels in livers of diet-induced obese mice treated with FGF1 for 14 hours.

Table S1

Gene	Forward 5'-3'	Reverse 5'-3'
hAcc	CTCATCCAAACAGAGGGAACATC	CATGGGTCATGCCATAGTGGT
m/rAcc	CCATCCAAACAGAGGGAACATC	CTACATGAGTCATGCCATAGTGGT T
mApoB	GCCCATTGTGGACAAGTTGATC	CCAGGACTTGGAGGTCTTGGA
mApoCI	GGGCAGCCATTGAACATATCA	TTGCCAAATGCCTCTGAGAAC
mApoCII	TTACTGGACCTCTGCCAAGGA	CCCTGAGTTTCTCATCCATGC
mApoCIII	CCAAGACGGTCCAGGATGC	ACTTGCTCCAGTAGCCTTTCAGG
mbKlotho	ACACAACCTGATCAAGGCACA	CCTTCTGATGAGGGCGGAAG
mCct	ACCTGGCCCTAATGGAGCA	TTGGAGCTGGCTGCCGTAAACC
mCD36	GATCGGAACTGTGGGCTCAT	GGTTCCTTCTTCAAGGACAACTTC
hChop	GGAAATGAAGAGGAAGAATCAAAAAT	GTTCTGGCTCCTCCTCAGTCA
mChop	CAGGAAACGAAGAGGAAGAATCA	GCTCCTCTGTCAGCCAAGCTA
rChop	CAGGAAACGAAGAGGAAGAATCA	TAGCTTGGCTGACTGAGG
mCptT1α	CTCAGTGGGAGCGACTCTTCA	GGCCTCTGTGGTACACGACAA
h/m/rDerlin	GGAGGCCAGCAGACTATTTATTC	-
h/rDerlin	GGAGGCCAGCAGACTATTTATTC	GGCCTTAAATCGTGTTCCAAACC
mDerlin	GGAGGCCAGCAGACTATTTATTC	CTTAAATCGCGTTCCGAACC
h/m/rEdem1	ATGAACACCTGGATTGACTC	-
h/mEdem1	ATGAACACCTGGATTGACTC	AGGCAGATGGCATCTTCCAC
rEdem1	ATGAACACCTGGATTGACTC	AGGCAGATGGCGTCTTCCAC
h/m/rErdj3	CGCTTCCAAATGACCCAGG	ATAAAGGGGTACTCCATGCC
hErp72	GATGTCTCTGGCTATCCCACC	GGACTGCTCGATCATGTAATC
mErp72	ACGCCACCGAACAGACAGAC	CCAGACTGCTCAATCATGTAG
rErp72	ACGCCACCGAACAGACAGAC	CCAGACTGCTCAACCATGTAG
mFatp4	CCAGACAAGGGTTTTACAGATAAGCT	ACCTGCTGTGCACCACAATG
mFatp5	GTGCTGATTGTGGATCCAGAC	GAATGTTCTCAGCTAGCAGCTTG
hFAS	GATGACATCGTCCATTCGTTTGT	CCATGCAGCTCAGCAGGTCTA
m/rFAS	GGCATCATTGGGCACTCCTT	GCTGCAAGCACAGCCTCTCT
mFGF21	ACGACCAAGACACTGAAGCC	TTGAGCTCCAGGAGACTTTCTG
hGadd34	GGCTCAAGCGCCCAGAAACC	AATGGACAGTGACCTTCTCG
mGadd34	CCCGAGATTCCTCTAAAAGC	CCAGACAGCAAGGAAATGG
rGadd34	ACCCTGAGATTCCTCTGAAGG	ATCTCGTGCAAACTGCTCC
hGRP78	TGGTGATCAAGATACAGGTGACCT	GTGTTCCTTGGAATCAGTTTGGT
mGRP78	TGGTGATCAGGATACAGGTGATCT	GGTACCACAGTGTTCCTTGGAA
rGRP78	TGGTGATCAAGATACAGGTGACCT	GGTGGGCACCACAGTGTTCC
h/m/rGrp94	CCTGAGAGAACTGATTTCAAATGC	TGTCTGTGACATGCAGCAGG
h/m/rGp78	TGGCTTTCATGGCTGCAGAG	TCCCTTCGTGGTTGAGGTCC
h/m/rHerp	CTCAAGGCCCACCTGAGCCG	CAGCTTCCCAGAATAAATTAACC
mLpcat3	GGCCTCTCAATTGCTTATTT	AGGATGAGGAACTGAAGCAC
mMttp	CAAGCTCACGTACTCCACTGAAG	TCATCATCACCATCAGGATTCCT
hPDI1	CAAAATCAAGCCCCACCTGAT	CTGTTTGCAGTGACCACACC
m/rPDI1	CAAGATCAAGCCCCACCTGAT	CTGCTTGCAGTGACCACACC
mPemt	AGGAGTCCAGAGTGACCACATTTC	AGGAGAGCAACCACGTAGAC
mPgc1α	GACCCCAGAGTCACCAAATGA	TTCCAGAGAGTTCCACACTTAAGG T
hXbp1	GCTGAAGAGGAGGCGGAAG	GTCCAGAATGCCCAACAGG

Table S2

Gene name	Uniprot	LOG10 p-value	LOG2 fold change	Tissue specificity	Location
Apcs	P12246	8,19	2,44	Liver	Blood
Ahsg	P29699	6,67	0,96	Liver	Blood
Serpinf2	Q61247	4,23	0,57	Liver	Blood
Apob	E9Q414	3,88	0,65	Liver	Blood
Serpina3n	Q91WP6	3,69	0,36	Liver	Blood
C5	P06684	3,65	-0,23	Liver	Blood
Serpina6	Q06770	3,48	-0,59	Liver	Blood
Ttr	P07309	3,44	-0,33	Brain, liver, retina	Blood
Fn1	P11276	3,12	0,26	Ductus deference, placenta	Blood
Gpx3	P46412	3,01	-0,43	Kidney, thyroid gland	Blood
Azgp1	Q64726	2,88	-0,44	Liver, salivary gland	Blood
Itih4	A6X935	2,67	0,38	Liver	Blood
Serpina3m	Q03734	2,65	0,33	Liver	Blood
Gsn	P13020	2,63	-0,35	Not specific	Blood
Mbl1	P39039	2,52	-0,42	Liver	Blood
Cd5l	Q9QWK4	2,48	-0,92	Lymphoid tissues	Blood
F5	O88783	2,39	0,79	Brain, liver, placenta	Blood
Saa4	P31532	2,28	-0,34	Liver	Blood
Hrg	Q9ESB3	2,21	-0,32	Liver	Blood
Fcn1	O70165	2,15	-0,48	Blood, bone marrow	Blood
Jchain	P01592	2,11	-0,69	Lymphoid tissues	Blood
Ig kappa chain V-VI	P01679	2,10	-0,68	Lymphoid tissues	Blood
Pglyrp2	Q8VCS0	2,05	-0,37	Liver	Blood
Ighm	P01872	1,97	-0,90	Lymphoid tissues	Blood
Actg1	P63260	1,93	-0,38	Not specific	Intracellular
Me1	P06801	1,80	-1,15	Not specific	Intracellular
Serpina1b	P22599	1,74	0,40	Liver	Blood
Ig heavy chain V region	P06330	1,72	-0,66	Lymphoid tissues	Blood
Adipoq	Q60994	1,64	-0,51	Adipose tissue, breast	Blood
Ig kappa chain V-V	P01645	1,51	-0,74	Lymphoid tissues	Blood
Ig heavy chain V-III	P01799	1,48	-0,73	Lymphoid tissues	Blood
Igkc	P01837	1,37	-0,61	Lymphoid tissues	Blood
Ig kappa chain V-III	P01656	1,31	-1,07	Lymphoid tissues	Blood
