# Supplementary material for: FGF1 ameliorates hepatic steatosis through acute activation of the unfolded protein response and VLDL production
Source: JHEP Rep. 2025 Oct 30;8(2):101660. doi: 10.1016/j.jhepr.2025.101660 (PMC12800356; doi:10.1016/j.jhepr.2025.101660)
Supplement: Multimedia component 2 [file mmc2.docx]

**JHEP Reports**

**CTAT methods**

Tables for a “Complete, Transparent, Accurate and Timely account” (CTAT) are now mandatory for all revised submissions. The aim is to enhance the reproducibility of methods.

- Only include the parts relevant to your study
- Refer to the CTAT in the main text as ‘Supplementary CTAT Table’
- Do not add subheadings
- Add as many rows as needed to include all information
- Only include one item per row

**If the CTAT form is not relevant to your study, please outline the reasons why:**

|  |
| --- |

- 1. **Antibodies**

| **Name** | **Citation** | **Supplier** | **Cat no.** | **Clone no.** |
| --- | --- | --- | --- | --- |
| Erp57 |  | Cell signaling, USA | 2887 |  |
| Erp72 |  | Cell signaling, USA | 2798 |  |
| Fatty Acid Synthase |  | Cell signaling, USA | 3189 |  |
| GRP78 |  | Cell signaling, USA | 3183 |  |
| GRP94 |  | Cell signaling, USA | 2104 |  |
| PDI |  | Cell signaling, USA | 2446 |  |
| Lamin A/C |  | Cell signaling, USA | 2032 |  |
| Phospho-4E-BP1 (Thr37/46) |  | Cell signaling, USA | 9459 |  |
| Phospho-4E-BP1 (Ser65) |  | Cell signaling, USA | 9451 |  |
| Phospho-Acetyl-CoA Carboxylase (Ser79) |  | Cell signaling, USA | 3661 |  |
| Acetyl-CoA Carboxylase |  | Cell signaling, USA | 3662 |  |
| Phospho-AKt (Ser473) |  | Cell signaling, USA | 9271 |  |
| Phospho-eEF2k (Ser366) |  | Cell signaling, USA | 3691 |  |
| Phospho-eIF2α (Ser51) |  | Cell signaling, USA | 9721 |  |
| Phospho-eIF4B (Ser422) |  | Cell signaling, USA | 3591 |  |
| Phospho-mTOR (Ser2448) |  | Cell signaling, USA | 2971 |  |
| Phospho-p44/42 MAPK (Erk1/2) (Thr202/Tyr204) |  | Cell signaling, USA | 9101 |  |
| p44/42 MAPK (Erk1/2) |  | Cell signaling, USA | 9102 |  |
| Phospho-p70 S6 Kinase (Thr389) |  | Cell signaling, USA | 9205 |  |
| p70 S6 kinase, Phospho-p90RSK (Thr359/Ser363) |  | Cell signaling, USA | 9344 |  |
| RSK1/RSK2/RSK3 |  | Cell signaling, USA | 9347 |  |
| Phospho-PERK (Thr980) |  | Cell signaling, USA | 12185 |  |
| Phospho-S6 ribosomal protein (Ser236 & Ser240) |  | Cell signaling, USA | 2215 |  |
| S6 ribosomal protein |  | Cell signaling, USA | 2217 |  |
| anti-ApoB |  | Meridian Bioscience, Cincinnati, USA | K23300R |  |
| anti-ATF6 |  | Active Motif, Carlsbad, USA | 40962 |  |
| anti-CHOP |  | Santa Cruz | Sc-7351 |  |
| anti-phospho-Ire1 (Ser724) |  | Abcam, Cambridge, UK | Ab48187 |  |
| anti-GAPDH |  | Calbiochem, San Diego, USA | CB1001 |  |

- 1. **Cell lines**

| **Name** | **Citation** | **Supplier** | **Cat no.** | **Passage no.** | **Authentication test method** |
| --- | --- | --- | --- | --- | --- |
| HepG2 |  | ATCC | HB-8065 | #2-20 | STR profiling |
| Hepa1-6 |  | ATCC | CRL-1830 | #2-20 | STR profiling |

- 1. **Organisms**

| **Name** | **Citation** | **Supplier** | **Strain** | **Sex** | **Age** | **Overall n number** |
| --- | --- | --- | --- | --- | --- | --- |
| *ob/ob mice* |  | Harlan | C57BL/6J | m | 8-12 weeks | 94 |
| *Wild type mice* |  | Harlan | C57BL/6J | m | 8-12 weeks | 40 |

- 1. **Sequence based reagents**

| **Name** | **Sequence** | **Supplier** |
| --- | --- | --- |
| N/A |  |  |

- 1. **Biological samples**

| **Description** | **Source** | **Identifier** |
| --- | --- | --- |
| N/A |  |  |

- 1. **Deposited data**

| **Name of repository** | **Identifier** | **Link** |
| --- | --- | --- |
| N/A |  |  |

- 1. **Software**

| **Software name** | **Manufacturer** | **Version** |
| --- | --- | --- |
| GraphPad Prism | GraphPad Software, San Diego, CA, USA | 8.00 |

- 1. **Other (*e.g*. drugs, proteins, vectors etc.)**

| FGF1 | R&D Systems |  |
| --- | --- | --- |
| FGF1 (K133E) | Novo Nordisk |  |

- 1. **Please provide the details of the corresponding methods author for the manuscript:**

| Tim van Zutphen, t.van.zutphen@umcg.nl |
| --- |

**2.0 Please confirm for randomised controlled trials all versions of the clinical protocol are included in the submission. These will be published online as supplementary information.**

| N/A |
| --- |
