# Supplementary material for: FGF1 ameliorates hepatic steatosis through acute activation of the unfolded protein response and VLDL production
Source: JHEP Rep. 2025 Oct 30;8(2):101660. doi: 10.1016/j.jhepr.2025.101660 (PMC12800356; doi:10.1016/j.jhepr.2025.101660)
Supplement: Multimedia component 4 [file mmc4.pdf]

Research article
MASLD and alcohol-related liver disease

JHEP|Reports

FGF1 ameliorates hepatic steatosis through acute activation of the unfolded protein response and VLDL production

Authors

Tim van Zutphen, Dicky Struik, Weilin Liu, …, Michael Downes, Ronald M. Evans, Johan W. Jonker

Correspondence

j.w.jonker@umcg.nl (J.W. Jonker).

Graphical abstract

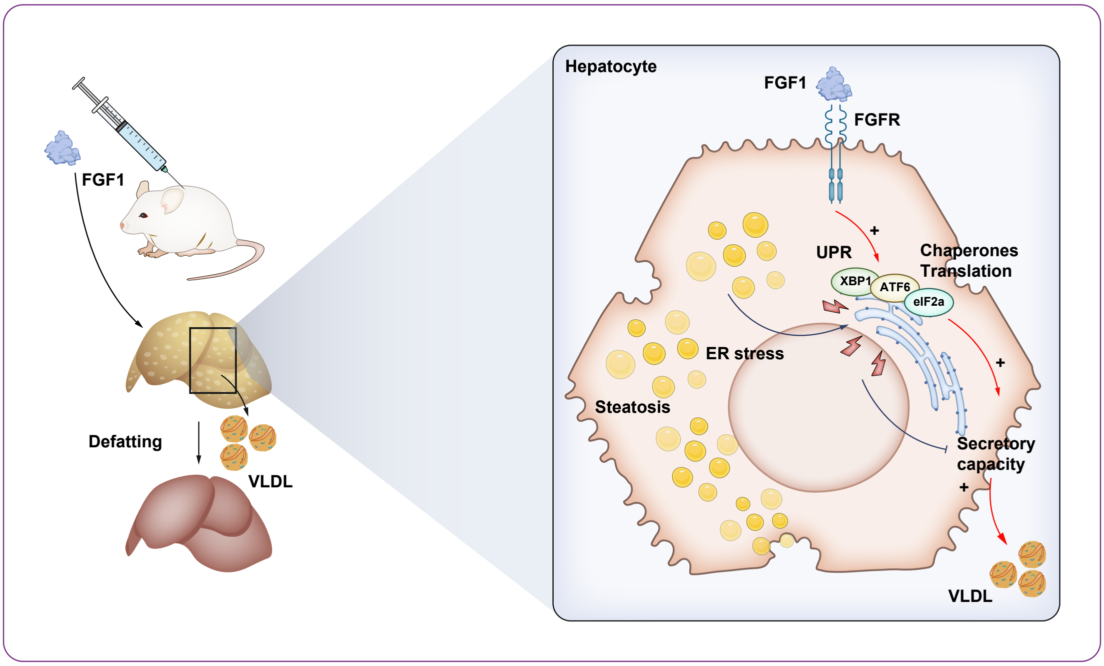

Highlights:

- FGF1 reduces hepatic fat accumulation by acutely driving VLDL secretion.
- FGF1 induces an adaptive unfolded protein response.
- The anti-steatotic action of FGF1 is potentiated by ER stress.

Impact and implications:

Fibroblast growth factors show therapeutic potential in both preclinical models and clinical trials for treating metabolic dysfunction-associated steatotic liver disease, a highly prevalent condition with limited treatment options. Identifying the mechanisms underlying their anti-steatotic effects may accelerate clinical development. Our finding that triglyceride secretion is the major driver of the anti-steatotic action of FGF1, together with the involvement of an adaptive unfolded protein response, provides deeper insight into the therapeutic potential of this pathway. These results also highlight possible implications for liver physiology and for the circulating lipoprotein profile, with relevance for both efficacy and safety considerations.

https://doi.org/10.1016/j.jhepr.2025.101660
© 2025 The Author(s). Published by Elsevier B.V. on behalf of European Association for the Study of the Liver (EASL). This is an open access article under the CC BY license (http://creativecommons.org/licenses/by/4.0/). JHEP Reports, 2026, 8, 1–13

JHEP|Reports

FGF1 ameliorates hepatic steatosis through acute activation of the unfolded protein response and VLDL production

Tim van Zutphen[1,2], Dicky Struik[1], Weilin Liu[1], Sihao Liu[3], Benan Pelin Sermikli[3], Justina C. Wolters[1], Henkjan J. Verkade[1], Annette R. Atkins[3], Michael Downes[3], Ronald M. Evans[3], Johan W. Jonker[1,*]

JHEP Reports **2026**. vol. 8 | 1–13

Check for updates

Background & Aims: Metabolic dysfunction-associated steatotic liver disease (MASLD) is a serious chronic liver disease with limited therapeutic options. Fibroblast growth factor (FGF) analogs show promising therapeutic benefits for MASLD, yet the underlying mechanisms remain incompletely understood. Here, we studied the mechanism underlying the anti-steatotic properties of FGF1, the prototype member of the FGF family.

Methods: The effect of FGF1 was studied in human and rodent hepatocytes and in obese mouse models exhibiting acute or chronic endoplasmic reticulum (ER) stress characteristic of MASLD. Metabolic analysis and proteomics were applied to evaluate liver physiology, ER stress and signaling.

Results: We show that FGF1 reduces hepatic triglyceride (TG) levels in obese mice (51%, p <0.01, n = 8) via acute stimulation of very-low-density lipoprotein (VLDL, 3.9-fold, p <0.01, n = 8) secretion in an ER stress-dependent manner. This anti-steatotic effect was independent of adipose FGF receptor 1, which is required for the glucose-lowering effect of FGF1. Mechanistically, activation of the unfolded protein response (UPR), resulting in stabilization of apolipoprotein B (ApoB, 1.8-fold, p <0.01, n = 8), the main structural protein component of atherogenic lipoprotein particles, was identified as the key mechanism by which FGF1 drives VLDL secretion. Post-translational control of ApoB by FGF1 was potentiated by pre-existing ER stress. FGF1 stimulated major regulators of protein synthesis, and during ER stress, all three branches of the UPR were activated. In ER stress-primed lean mice, FGF1 adopted novel TG secretion activity (2.2-fold, p <0.05, n = 6). Conversely, alleviation of ER stress in obese mice suppressed FGF1-stimulated VLDL-TG production (49%, n = 11, p <0.05).

Conclusion: These results define ER stress-dependent modulation of VLDL secretion as a mechanism underlying the anti-steatotic activity of FGF1. Targeting the FGF-UPR pathway may thus have therapeutic potential for treating MASLD.

© 2025 The Author(s). Published by Elsevier B.V. on behalf of European Association for the Study of the Liver (EASL). This is an open access article under the CC BY license (http://creativecommons.org/licenses/by/4.0/).

Introduction

Metabolic dysfunction-associated steatotic liver disease (MASLD) is the most common chronic liver disorder worldwide and encompasses a broad spectrum of liver conditions driven by excessive hepatic lipid accumulation.[1] Sustained steatosis predisposes to more severe conditions such as metabolic dysfunction-associated steatohepatitis (MASH), cirrhosis, and ultimately hepatocellular carcinoma and end-stage liver disease.[2] In addition, MASLD is considered an independent risk factor for cardiovascular disease and has become a major indication for liver transplantation.[2,3] MASLD has also been linked to other complications, such as chronic kidney disease and extrahepatic malignancies, particularly colorectal cancer.[4,5] The pathophysiological mechanisms underlying MASLD include insulin resistance, lipotoxicity, mitochondrial dysfunction, and perturbed endoplasmic reticulum (ER) proteostasis.[6] Despite a wealth of research and the considerable disease burden, treatment options for MASLD are limited. Resmetirom was approved very recently for advanced stages of MASH (by the FDA, while still under evaluation by the EMA) and Glucagon-like peptide-1 receptor agonists have shown potential in reducing both hepatic liver accumulation and MASH but not fibrosis.[7]

In the past decade, endocrine members of the fibroblast growth factor (FGF) family (*i.e.* FGF19 and FGF21) have been identified as critical regulators of metabolic homeostasis with strong anti-steatotic effects.[8,9] Several FGF analogs have shown promising results in clinical trials for MASLD/MASH.[10] Previously, we discovered that FGF1, a paracrine FGF family member, plays a crucial role in adipose function and that it is effective in the treatment of type 2 diabetes in mouse models.[11,12] Systemic administration of recombinant FGF1 results in various metabolic improvements, including acute normalization of blood glucose levels and a reduction in hepatic steatosis and inflammation upon prolonged treatment.[12–14] FGF1 can bind to all FGF receptors and acts independently of the FGF19/FGF21 coreceptor β-Klotho, which may be transcriptionally downregulated in adipose

* Corresponding author. Address: Department of Pediatrics, University of Groningen, University Medical Center Groningen, Groningen, The Netherlands.
E-mail address: j.w.jonker@umcg.nl (J.W. Jonker).
https://doi.org/10.1016/j.jhepr.2025.101660

tissue and the liver during obesity.[15,16] The glucose-lowering effects of FGF1 are mediated by phosphodiesterase 4D (PDE4D)-dependent suppression of adipose tissue lipolysis, which subsequently lowers hepatic glucose production.[17] While several independent research groups have reported the anti-steatotic actions of FGF1 treatment, the underlying mechanisms, especially those that play a role during the earliest phases of restoring metabolic homeostasis, remain unclear.[12,14,18,19] In this study, we explored the mechanisms that drive the anti-steatotic effects of FGF1.

Materials and methods

Animals

Animals used in this study were male wild-type and *ob/ob* mice on a C57BL/6J genetic background (Harlan, Zeist, The Netherlands), between 8-12 weeks of age. Animals were housed in a light- and temperature-controlled facility (lights on from 7 a.m. to 7 p.m., 21 °C). All mice received a standard laboratory chow (RMH-B; Hope Farms, Woerden, The Netherlands) and acidified water *ad libitum*, except diet-induced obese (DIO) mice that received a high-fat diet containing 60% kcals from fat (research diets D12492) for 16 weeks. During time-course experiments, mice received a low-fat diet. Mice were housed and handled according to institutional guidelines complying with Dutch legislation. All experiments were approved by the Ethical Committee for Animal Experiments, University of Groningen, The Netherlands.

Animal experiments

Mice received a standard chow diet (RM1; SDS Diets, Woerden, The Netherlands), unless otherwise indicated. Adipose-specific *Fgfr1*$^{-/-}$ mice and DIO mice received a high-fat diet (60% kcal from fat, Research Diets D12492). Mice were treated with vehicle (PBS), recombinant FGF1 (0.5 mg/kg, R&D Systems) or the FGF1 K133E variant (kind gift by Birgitte Andersen, Novo Nordisk, Bagsværd Denmark) by intraperitoneal (i.p.) injection. During chronic treatments, mice received an i.p. injection every 3 days for 2 or 5 weeks. Some mice received a pre-treatment with tunicamycin (0.1 mg/kg i.p., Sigma-Aldrich, St Louis, USA) or 4-phenylbutyrate via the drinking water (500 mg/kg, assuming 5 ml/day intake of a 4 g/L solution). Poloxamer 407 (P-407) 1 mg/kg was injected i.p. For indirect calorimetry measurements, mice were placed in an open-circuit indirect calorimeter system for 24 h prior to the start of the experiment with free access to water and food (TSE systems GmbH, Bad Homburg, Germany). O_2 and CO_2 flow rates were measured and analyzed using LabMaster software (TSE systems GmbH, Bad Homburg, Germany) to produce VO_2, VCO_2, and the respiratory exchange ratio: VCO_2/VO_2. Mice were euthanized by cardiac puncture after anesthesia with isoflurane. Terminal blood samples were collected in EDTA-coated tubes. Tissues were collected and frozen in liquid nitrogen.

Proteomics sample preparation

Protein levels were determined using unbiased proteomics (using label-free quantification) for relative protein concentrations[20] or targeted proteomics (using an isotopically labeled standard for lipid metabolic proteins) to accurately quantify a subset of metabolic proteins.[21] Briefly, in-gel digestion was performed on 1 µl plasma using trypsin (1:100 g/g sequencing grade modified trypsin V5111; Promega) after reduction with 10 mmol/L dithiothreitol and alkylation with 55 mmol/L iodoacetamide proteins, followed by solid-phase extraction (SPE C18-Aq 50 mg/1 ml, Gracepure, Thermo Fisher Scientific) for sample clean-up.

Untargeted proteomics

Unbiased mass spectrometric analyses were performed on a quadrupole orbitrap mass spectrometer equipped with a nano-electrospray ion source (Orbitrap Q Exactive Plus, Thermo Scientific). Chromatographic separation of the peptides was performed by liquid chromatography (LC) on a nano-HPLC system (Ultimate 3000, Dionex) using a nano-LC column (Acclaim PepMapC100 C18, 75 µm x 50 cm, 2 µm, 100 Å, Dionex, buffer A: 0.1% v/v formic acid, dissolved in milliQ-H_2O, buffer B: 0.1% v/v formic acid, dissolved in acetonitrile). 50 nl digested plasma was injected using the µl-pickup method with buffer A as a transport liquid from a cooled autosampler (5 °C) and loaded onto a trap column (µPrecolumn cartridge, Acclaim PepMap100 C18, 5 µm, 100 Å, 300 µmx5 mm, Dionex). Peptides were separated on the nano-LC column using a linear gradient from 2-45% buffer B (117 min, flow rate 200 nl/min). The mass spectrometer was operated in positive ion mode and data-dependent acquisition mode using a top-15 method. Mass spectrometry (MS) spectra were acquired at a resolution of 70,000 at m/z 200 over a scan range of 300 to 1,650 m/z with an AGC target of 3×10^6 ions and a maximum injection time of 50 ms. Peptide fragmentation was performed with higher energy collision dissociation using normalized collision energy of 28. The intensity threshold for ion selection was set at 2.0×10^4 with a charge exclusion of 1≤ and ≥7. The MS/MS spectra were acquired at a resolution of 17,500 at m/z 200, an AGC target of 1×10^5 ions, a maximum injection time of 50 ms, and the isolation window set to 1.8 m/z.

LC-MS raw data were processed with MaxQuant (version 1.6.12.0).[22] Peptide and protein identification were carried out with Andromeda against a mouse SwissProt database (www.uniprot.org, downloaded 1912, 17021 entries). The searches were performed using the following parameters: precursor mass tolerance was set to 20 ppm, for peptide identification, two miss cleavages were allowed, a carbamidomethylation on cysteine residues as a static modification, and oxidation of methionine residues and an acetyl modification on the N-terminus as variable modifications. Peptides and proteins were identified with an false discovery rate of 1%. Proteins were quantified with the MaxLFQ algorithm, including both razor and unique peptides and a minimum ratio count of one.[22] The results were imported into Perseus (version 1.5.5.3[23]) for further processing and filtering. Protein groups were filtered for proteins quantified in four out of the six replicates in both groups.

Targeted proteomics

LC on a nano-ultra high-performance LC system (Ultimate UHPLC focused; Dionex, Thermo Fisher Scientific) was performed to separate the peptides using a nanocolumn (Acclaim PepMap100 C18, 75 µm × 500 mm 2 µm, 100 Å) with a linear gradient from 3% to 40% v/v acetonitrile plus 0.1% v/v formic acid in 90 min at a flow rate of 200 nl/min. The target peptides were analyzed by a triple quadrupole MS equipped with a

nano-electrospray ion source (TSQ Vantage; Thermo Scientific), as described previously for a subset of the targets.[21] For the LC-MS measurements, an amount of the digested peptides equivalent to a total protein amount of 50 ng total protein starting material was injected with 1.5 ng isotopically labeled concatemer-derived standard peptides (QconCAT technology, PolyQuant GmbH Germany). The peptides used for the quantification of the target proteins are listed in Table 1. The MS traces were manually curated using the Skyline software before integrating the peak areas for quantification. The sum of all transition peak areas for the endogenous peptide and isotopically labeled QconCAT-peptide standard was used to calculate the endogenous and standard peptide ratio. The concentrations of the endogenous peptides were calculated from the known concentration of the standard and expressed in mg/dl plasma starting material. The average of two peptides was used for proteins targeted with multiple peptides.

Biochemical analysis

Analysis of plasma alanine aminotransferase (ALT) (Spinreact, Girona, Spain), Albumin (Sigma-Aldrich), free fatty acids (Dia-Sys, Germany, 15781), total cholesterol (Roche, Mijdrecht, The Netherlands 11491458-216) and triglycerides (TGs) (Roche, Mijdrecht, The Netherlands, cat.no 1187771216) was performed using commercial kits according to the manufacturer's instructions. For fractionation of lipoprotein particles, plasma was pooled and subjected to fast protein liquid chromatography (FPLC) gel filtration using a Superose 6 column (GE Healthcare, Uppsala, Sweden). Extraction of lipids from hepatocytes or liver was performed according to established procedures.[24] 0.4 µCi ^{14}C-palmitate (Perkin Elmer, Waltham, USA) conjugated to BSA was supplemented to hepatocytes at the start of the treatment. Hepatic TG levels were measured with a commercial kit according to the manufacturer's instructions (Roche, Mannheim, Germany). Lipids were separated on thin-layer chromatography plates (Merck Millipore, Darmstadt, Germany) and analyzed for radioactivity using a 2D plate reader (Berthold Tracemaster, Wildbad, Germany). Plasma acylcarnitine profiles were measured by LC-MS/MS. Commercially available ELISA kits were used to determine plasma insulin (Chrystal Chem, USA, 90010), mouse total bile acids (Chrystal Chem, USA, 80470), and glucagon (Chrystal Chem, USA, 81518).

Fatty acid β-oxidation

Freshly harvested cells were homogenized and centrifuged at 500 rpm for 10 min, after which the supernatant containing crude mitochondria was incubated with 0.4 µCi ^{14}C palmitate conjugated to BSA, followed by trapping of radiolabeled CO_2 and scintillation counting (Packard, 1600Ca Tri-Carb, Meriden, USA).[25]

Acylcarnitines were extracted from plasma and liver tissue homogenates using acetonitrile and analyzed by mass spectrometry.

Table 1. List of peptides used to target apolipoproteins.

Protein	Peptide
APOA1	DFWDNLEK
APOA4	ALVQQLEQFR
APOB	QSFDLSVK
APOB	VQGVEFSHR
APOC1	EFGNTLEDK
APOC1	AWFSEAFGK
APOC2	SSAAMSTYAGIFTDQLLTLLR
APOC2	TYPISMDEK
APOC3	TVQDALSSVQESDIAVVAR
APOE	FWDYLR
APOM	FLLYNR

Lipogenesis

1-^{13}C-acetate supplemented drinking water (2% w/v) was given *ad libitum* to mice 24 h before termination. Liver homogenates were incubated in methanol/HCl at 90 °C for 4 h, followed by fatty acid extraction using hexane and analysis of label incorporation into fatty acids by gas chromatography MS.[26]

VLDL secretion

To assess the very low-density lipoprotein (VLDL) secretion rate, Poloxamer 407 (P-407) 1 mg/kg was injected i.p. and TG concentrations were assessed at different time points. From the slope of the curves, using T = 30, 60 and 180 min, the VLDL-TG production rates were calculated as described.[26]

Cell culture

Primary hepatocytes were isolated from 200 to 250 g male Wistar rats using a two-step perfusion method,[27] without using growth factors during the attachment period and with 50 nmol/L dexamethasone (Sigma-Aldrich, Saint-Louis, USA). HepG2 and Hepa1-6 cells were obtained from ATCC and cultured according to standard procedures. Before the experiments, serum was removed overnight. Replicates were derived from individual isolations or separate culture passages. Hepatocytes received 100 ng/ml FGF1 (CYT-528, Prospec, Israel) or PBS as control. To assess the effect of FGF1 under stress conditions, cells were pre-treated to induce ER stress for 2 h prior to FGF1 administration.

Protein analysis

For immunoblot analysis, whole-cell lysates were prepared in RIPA lysis buffer (Tris-HCL pH = 8, NaCl 138 mM, NP40 1%, KCl 2.7 mM, $MgCl_2$ 1 mM, Glycerol 5%, EDTA 5 mM, Na_3VO_4 1 mM, NaF 20 mM, DTT 1 mM and protease inhibitor) and protein concentrations were quantified using the RC DC assay (Bio-Rad Hercules, USA). Protein samples were subjected to SDS-PAGE (15% gels) and transferred to nitrocellulose using the Trans-Blot® TurboTM transfer system (Bio-Rad). After blocking for 1 h at room temperature in PBS containing 0.1% Tween and 2% milk powder, membranes were incubated overnight with primary antibodies at 4 °C. Antibodies used in this study are described in the CTAT table. Antibodies were detected by incubating the blot with horseradish peroxidase-conjugated donkey anti-rabbit (Life science, NA934) or rabbit anti-mouse (Dako, p0260) IgG for 1 h at room temperature. Bands were visualized using Supersignal West Dura substrate (Thermo Scientific, Waltham, USA) and ChemiDoc (Bio-Rad). Image Lab software (Bio-Rad) was used for densitometry.

Gene expression analysis

Total RNA was isolated from the liver using Tri reagent (Life Technologies, USA) and reverse transcribed into cDNA using M-MLV, random primers, and dNTPs according to standard procedures. For quantitative PCR (qPCR), cDNA was amplified using Hi-ROX SensiMix^{TM} SYBR green (Bioline, London, UK) and StepOnePlus^{TM} Real-Time PCR System (Applied Biosystems, CA, USA). Primers used for qPCR are listed in Table S1. U36B4 was used as the housekeeping gene in all PCR analyses, and the ΔΔCt method was used for quantification.

Statistical analysis

Statistical analyses were performed using the GraphPad Prism 8.00 software package (GraphPad Software, San Diego, CA, USA). Significance was determined using the non-parametric Mann-Whitney *U* test when comparing two groups or the Kruskal-Wallis test with Dunn's *post hoc* test when comparing more groups. All values are given as means ± SD unless stated otherwise. Significance was indicated as **p* <0.05, ***p* <0.01, ****p* <0.001.

Results

Identification of VLDL as a metabolic target of FGF1

Previously, we have reported strong anti-steatotic effects of FGF1.[12] To confirm these findings in our current study, we treated *ob/ob* mice with recombinant FGF1 every 3 days for 2 weeks. As expected, this treatment resulted in a two-fold reduction in hepatic TG levels compared to those in saline-treated controls (Fig. S1A). We have previously demonstrated that the glucose-lowering properties are dependent on adipose FGF receptor 1 (FGFR1).[12] To test the potential involvement of adipose FGFR1 on the anti-steatotic properties of FGF1, obese, adipose-specific *Fgfr1*$^{-/-}$ mice were administered FGF1 every 3 days for 5 weeks. Hepatic TG levels were strongly reduced compared to saline-treated controls, indicating that the anti-steatotic effect of FGF1 is independent of adipose FGFR1 and not driven by suppression of lipolysis or glucose-lowering (Fig. 1A). Accordingly, free fatty acid plasma levels (Fig. 1B) and the expression levels of hepatic fatty acid transporter genes CD36, fatty acid transport protein 4 (*Fatp4*) and 5 (*Fatp5*) were not affected (Fig. S1B). Interestingly, a marked increase in plasma TG levels 24 h after an FGF1 injection was observed in *ob/ob* mice, pointing towards hepatic TG secretion as an underlying mechanism (Fig. 1C). In contrast, total plasma cholesterol levels remained similar to controls (Fig. 1D).

To assess whether FGF1's anti-steatotic action is mediated via acute TG secretion, we performed untargeted plasma proteomics in ob/ob mice 24 h after a single FGF1 injection. Out of 132 identified proteins, 33 (25%) were significantly affected by FGF1 treatment. Twenty-three proteins were decreased, and 10 proteins were increased (Fig. 1E, Table S2). About half (52%) of all the plasma proteins affected by FGF1 were known or predicted to be of hepatic origin (Table S2). In line with the elevated circulating TG levels after a single FGF1 injection, apolipoprotein B (ApoB) was identified among the most strongly elevated proteins (Fig. 1E, arrow). ApoB forms the structural scaffold for chylomicrons, VLDL, LDL, and intermediate-density lipoprotein particles, all of which are important for TG and cholesterol transport.[28]

To confirm the effect of FGF1 on ApoB and to examine whether other apolipoproteins were also affected, targeted proteomics of lipoproteins was performed, replicating an increase in plasma ApoB100 levels after FGF1 administration to *ob/ob* mice (Fig. 1F). In addition to ApoB100, FGF1 also significantly increased ApoC2 and ApoM concentrations, while plasma levels of ApoA1, ApoA4, ApoC1, ApoC3, and ApoE remained unaffected (Fig. 1F). The single FGF1 treatment did not significantly alter hepatic mRNA levels of apolipoprotein genes, suggesting that FGF1 modulates apolipoprotein levels mainly at the post-translational level (Fig. S1C).

FGF1 stimulates hepatic VLDL secretion in *ob/ob* mice

Next, the temporal dynamics of plasma TG levels after FGF1 injection were evaluated. Time-course analysis in *ob/ob* mice after a single FGF1 injection showed a robust and transient increase in plasma TG levels that returned to baseline levels after approximately 48 h (Fig. 2A). In addition, 24 h after the last FGF1 injection of a chronic 2-week treatment regimen, which reduced hepatic steatosis by approximately 50% (data not shown), plasma TG levels were increased to a similar extent compared to a single acute injection, indicating that adaptation to FGF1 treatment does not develop within this time frame (Fig. S2A). FPLC confirmed that FGF1 strongly increased plasma TGs in ApoB-containing lipoproteins, mainly in the VLDL fraction (≈4-fold) and to a smaller extent in the LDL fraction (≈2-fold, Fig. 2B). Immunoblotting of the VLDL fractions showed that the increase in plasma TG levels was accompanied by an increase in ApoB protein levels (Fig. 2B, inset). The increase in both TG and ApoB protein levels by FGF1 resulted in similar TG/ApoB100 ratios compared to controls, indicating that lipidation of ApoB was not affected by FGF1 (Fig. 2C). Hepatic mRNA levels of *MTTP* (microsomal triglyceride transfer protein), encoding the enzyme responsible for ApoB lipidation, were also unaffected (Fig. S2B). In line with the unaltered FPLC elution of VLDL fractions and TG/ApoB100 ratios after FGF1 treatment, the plasma TG to phospholipid (TG/PL) ratio also remained similar to controls, further confirming that VLDL particle size was not substantially affected (Fig. S2C).

As increased plasma VLDL levels can result from decreased VLDL clearance as well as increased VLDL production, *ob/ob* mice were treated with the lipoprotein lipase inhibitor poloxamer 407 to determine the effect of FGF1 on the VLDL synthesis rate. As shown in Fig. 2D, FGF1 significantly increased the VLDL-TG secretion rate (indicated in the right panel) when lipoprotein lipase, and thus VLDL clearance, was blocked, indicating that FGF1 acutely increased plasma VLDL levels by stimulating its secretion. A single FGF1 treatment was not associated with changes in plasma levels of known regulators of VLDL metabolism, including insulin, glucagon, or bile acids (Fig. S2D-F). However, similar to FGF19, FGF1 suppressed the expression of the rate-limiting enzyme in bile acid synthesis, *Cyp7A1*, both in the liver and hepatocytes (Fig. S2G).[29] As expected, FGF1 stimulated the expression of genes associated with proliferation (Fig. S2H). However, since a non-mitogenic FGF1 variant (K133E) was also able to increase plasma TG levels (Fig. 2E), this suggests that the effects of

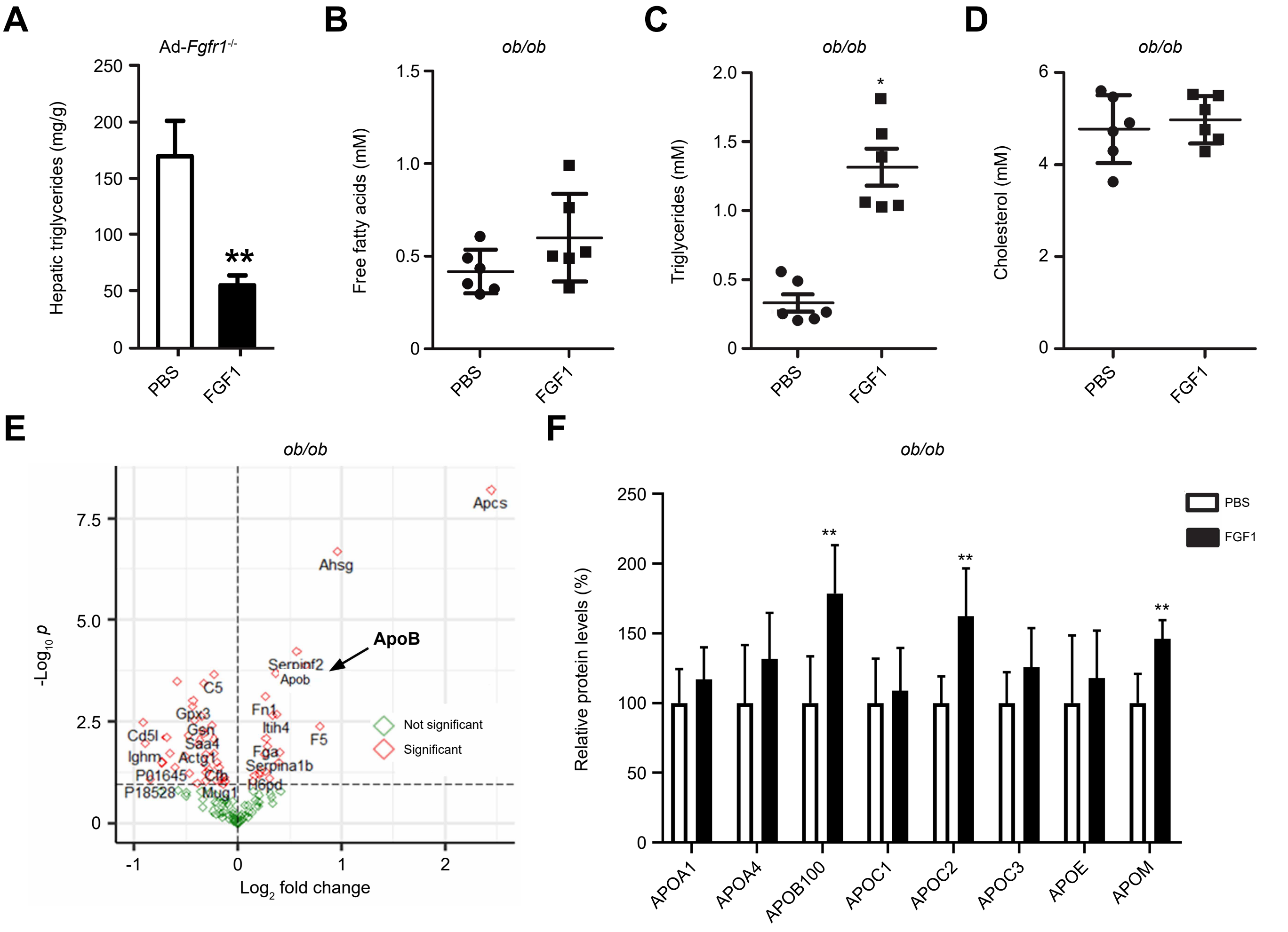

Fig. 1. FGF1 reduces hepatic steatosis and acutely induces ApoB. (A) Hepatic TG in high-fat diet-fed adipose-specific *Fgfr1*$^{-/-}$ mice treated with FGF1 every 3 days for 5 weeks (Mann-Whitney *U* test; ***p* <0.01). (B) Plasma free fatty acid levels, (C) plasma TG, and (D) total cholesterol levels 24 h after an FGF1 injection in chow-fed *ob/ob* mice (Mann-Whitney *U* test; **p* <0.05). (E) Volcano plot of significantly (red) and non-significantly (green) affected plasma proteins in chow-fed *ob/ob* mice after 24 h FGF1 treatment as determined by untargeted proteomics. Arrow highlights ApoB. (F) Profiling of apolipoproteins in plasma of chow-fed *ob/ob* mice after 24 h of FGF1 treatment using a targeted proteomics strategy (Mann-Whitney *U* test; ***p* <0.01). All panels n = 6-8. TG, triglyceride.

FGF1 on VLDL secretion are independent of its mitogenic effects.

The acute effects of FGF1 on hepatic lipid metabolism are limited to TG efflux

Subsequently, we determined if modulation of intracellular hepatic lipid fluxes contributes to the acute anti-steatotic effects of FGF1. Hepatic lipogenesis fluxes were quantified using a stable isotope-labeled fatty acid precursor to evaluate acute modulation of lipid synthesis pathways. A single dose of FGF1 did not affect the fractional lipid synthesis rate (lipogenesis) as measured by the accumulation of ^{13}C-acetate over 24 h in the main fatty acid species palmitate (C16:0), palmitoleate (C16:1), stearate (C18:0), and oleate (C18:1), nor did FGF1 affect the chain elongation of pre-existing C16 to C18 species in the livers of *ob/ob* mice (Fig. 3A). In line with these observations, no changes were observed in the mRNA levels of the lipogenic enzymes acetyl-CoA carboxylase or fatty acid synthase in hepatocytes (primary rat hepatocytes or HepG2 cells after 6 h, Fig. S3A), nor their protein levels in *ob/ob* mouse livers (Fig. 3B). Phosphorylation of acetyl-CoA carboxylase at the AMPK-responsive Ser-79 was also not affected after a single FGF1 administration (Fig. 3B).

Hepatic acylcarnitine levels also remained unaltered 24 h after a single injection, indicating that fatty acid β-oxidation was not strongly affected, as was also observed previously (Fig. 3C: C14–C18, Fig. S3B: full spectrum).[17] Similarly, FGF1 did not increase β-oxidation rates in the hepatocyte cell lines HepG2 and Hepa1-6 (Fig. S3C). In addition, a single FGF1 treatment did not alter hepatic mRNA levels of the fatty acid catabolic genes *Cpt1a* and *Pgc1α* in *ob/ob* mice (Fig. S3D). However, unlike hepatic long-chain acylcarnitines, plasma levels of long-chain acylcarnitines were elevated after FGF1 treatment (Fig. 3D: C14–C18, Fig. S3E: whole spectrum). Whole-body analysis of substrate utilization using indirect calorimetry also showed a clear yet transient shift towards lipids as a predominant fuel source upon a single FGF1 injection in *ob/ob* mice (Fig. 3E). The observed switch to lipids as a fuel source, together with the elevation of plasma

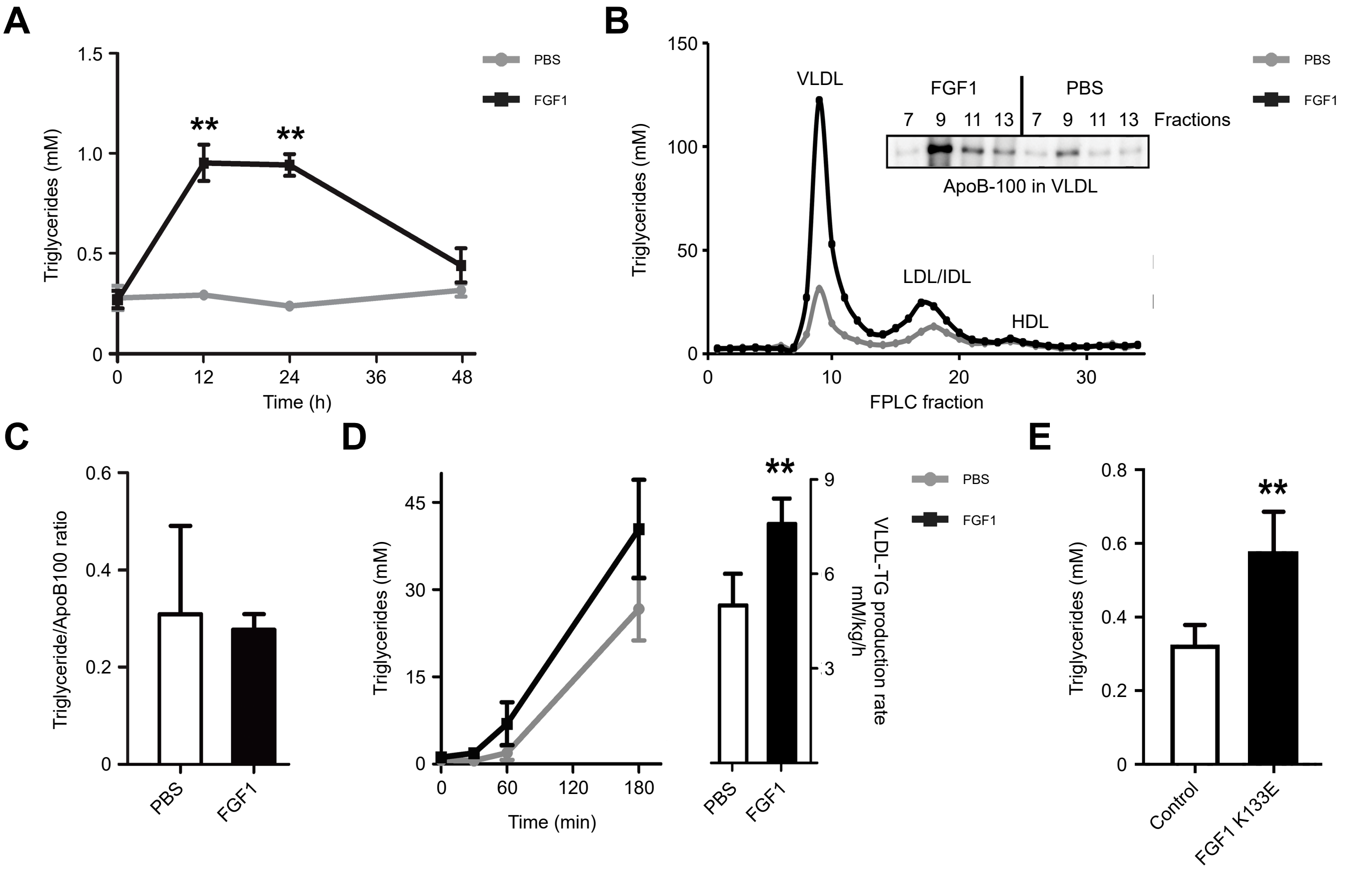

Fig. 2. FGF1 acutely and transiently stimulates VLDL production in *ob/ob* mice. (A) Temporal dynamics of plasma TG in *ob/ob* mice upon FGF1 injection (Mann-Whitney *U* test; ***p* <0.01). (B) TG levels in FPLC fractions from plasma of *ob/ob* mice, 24 h after FGF1 injection. Inset: ApoB in FPLC fractions 7, 9, 11 and 13 corresponding to VLDL. (C) Plasma TG/ApoB ratios 24 h after an FGF1 injection in *ob/ob*. (D) Left panel: plasma TG levels upon LPL inhibition by poloxamer in 14 h FGF1-stimulated *ob/ob* and right panel: calculated production rate from the curves. (E) Plasma TG in *ob/ob* mice 24 h after FGF1 K133E administration (Mann-Whitney *U* test; ***p* <0.01). All panels n = 6-8. FPLC, fast protein liquid chromatography; TG, triglyceride.

acylcarnitines while hepatic acylcarnitines remain unaffected, indicates that extrahepatic tissues likely provide a sink for the TGs liberated from the liver.

As FGF1 appears to specifically affect VLDL production rates without acutely modulating other major hepatic lipid fluxes, we postulate that FGF1 primarily regulates TG secretion, thereby clearing hepatic steatosis in obese mice.

FGF1 activates the unfolded protein response pathway and major regulators of protein synthesis

Given that FGF1 increased ApoB100 protein levels in plasma without affecting its transcription or lipidation (see Fig. 1E and Fig. S1A), regulation of VLDL synthesis at the post-translational level may occur. VLDL synthesis is initiated by the production of ApoB100 in the ER. As one of the largest monomeric proteins with a highly hydrophobic core, ApoB100 folding is highly sensitive to ER stress, a phenomenon that is safeguarded by an adaptive process called the unfolded protein response (UPR).[6] Based on this we wondered if FGF1 acts by modulating the UPR. A single dose of FGF1 indeed resulted in potent activation of all three UPR branches in livers of *ob/ob* mice, assessed by increased Xbp1 protein, spliced Atf6, and increased phosphorylation of Perk (Fig. 4A). FGF1 also increased nuclear localization of Xbp1 and Atf6, confirming full pathway activation (Fig. 4A). Activation of the UPR sensors was accompanied by induction of the downstream ER chaperones at the level of mRNA and protein (Fig. 4B,C). Similar to plasma levels, hepatic protein levels of ApoB were also increased, while MTTP levels were not. In contrast to the chaperones, ER-associated degradation components were not affected by FGF1 and neither were Chop and GADD34 (Fig. 4B). After prolonged FGF1 treatment (*i.e.* for 2 weeks), the hepatic UPR was still evident, indicating that consecutive FGF1 injections do not dampen the response (Fig. S4A). The livers of these mice showed an approximately two-fold reduction in hepatic TG levels (Fig. S1A) and accordingly, hepatic Fgf21 expression decreased after treatment, whereas expression of the FGF coreceptor β-klotho remained unaltered (Fig. S4B).[30]

To establish whether FGF1 activates the UPR via a direct effect on hepatocytes, we examined the potential of FGF1 to activate the UPR in primary hepatocytes and hepatocyte cell lines. Although FGF1 was not able to induce any of the common UPR markers in the human and murine hepatocyte cell lines HepG2 and Hepa1-6, or primary rat hepatocytes under standard conditions, UPR hyperactivation was observed when these cells were primed with ER stressors (Fig. 4D and Fig. S4C,D). In these ER-stressed hepatocytes, FGF1 caused

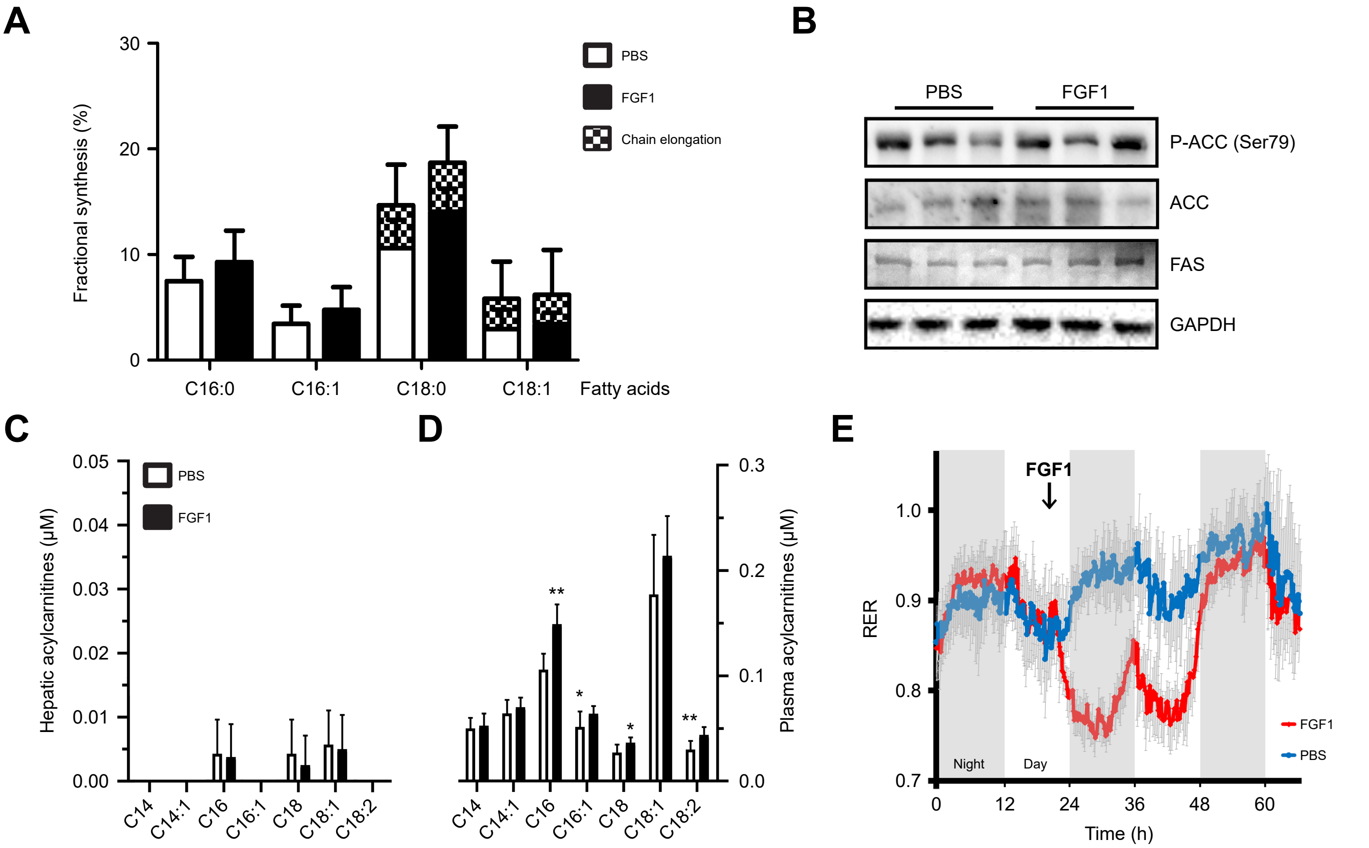

Fig. 3. Anti-steatotic FGF1 effects are not associated with adipose lipolysis, hepatic lipogenesis or β-oxidation. (A) Fatty acid synthesis and elongation rates of most common fatty acids using ^{13}C-acetate in *ob/ob* mouse livers during 24 h of FGF1 treatment. (B) Hepatic levels of lipogenesis proteins ACC and FAS, as well as phosphorylation of ACC in *ob/ob* mice 14 h after FGF1 injection. (C) Hepatic long-chain (C14–C18) acylcarnitines and (D) plasma acylcarnitines of 14 h FGF1-treated *ob/ob* mice. (E) Respiratory exchange ratios of FGF1-treated (at arrow) *ob/ob* mice. Mann-Whitney *U* test; **p* <0.05, ***p* <0.01; n = 6-8.

hyperactivation of all three UPR branches, including splicing of *Xbp1* RNA and increased Xbp1s protein, activation of the upstream kinase Ire1, phosphorylation of Perk, and an increase in spliced, active ATF6 (Fig. 4D). Also, FGF1 increased mRNA and protein levels of ER chaperones, including Grp78, Erp72, and Erdj3 (Fig. 4E,F and Fig. S4D). In contrast, mRNA levels of ER-associated degradation components involved in the clearance of ER-luminal polypeptides – including *Gp78*, *Derlin*, *Edem,* and *Herp* – were not affected by FGF1. Except for its direct target eIF2α, downstream targets of PERK such as Atf4, Chop, and Trib3 were also unchanged (Fig. 4E and Fig. S4E,F).

Together, these findings indicate that FGF1 stimulates all three branches of the UPR in hepatocytes and the liver, an effect that is potentiated by pre-existing ER stress.

As FGF1 has been shown to induce protein translation,[31] we considered stimulation of global protein synthesis and subsequent ER protein overload as a potential classical upstream signal for the UPR in hepatocytes. Indeed, in HepG2 cells and primary rat hepatocytes, activation of the major signaling cascades that regulate protein synthesis was observed upon stimulation by FGF1, including phosphorylation of the p90 and P70 ribosomal S6 kinases (P90RSK and p70S6K, respectively) as well as the downstream substrates eukaryotic elongation factor 2 kinase and the ribosomal subunit S6 (Fig. 5A). Besides P70S6K, mTORC1 itself, as well as another target, 4E-BP1 (eukaryotic translation initiation factor 4E-binding protein 1), showed marked but transient phosphorylation in HepG2 cells and primary rat hepatocytes, indicative of mTORC1 activation (Fig. 5A). In addition, Akt, acting upstream of mTORC1, was transiently phosphorylated upon FGF1 stimulation and these effects on Akt, mTORC1 and its downstream targets could be prevented by inhibiting various key components in this pathway in both HepG2 and Hepa1-6 cells (Fig. S5A,B).

To corroborate these findings *in vivo*, DIO mice were injected with FGF1, and livers were analyzed 15 min after FGF1 administration. In line with the findings in cell lines, increased phosphorylation of fibroblast growth factor receptor substrate 2a (FRS2a), 4E-BP1, P70S6K, P90RSK, and ribosomal subunit S6 at both Ser-236 and Ser-240 was observed in FGF1-treated livers compared to controls (Fig. 5B), indicating that FGF1 activates two major regulatory pathways of protein synthesis, through mTORC1 and P90RSK.

Next, we tested the contribution of these kinase signaling cascades to FGF1- dependent UPR induction *in vitro.* Inhibition of PI3K or mTORC effectively prevented the upregulation of ER chaperones Grp78, Erp72, and Erdj3 by FGF1 in glucosamine-treated HepG2 cells and primary rat hepatocytes (Fig. 5C and Fig. S5C,D). Inhibition of protein translation (by cycloheximide) or ER protein import (by eeyarestatin) had a similar effect. In addition, FGF1-induced UPR activation was prevented by increasing ER folding capacity using the chemical chaperone phenylbutyrate (Fig. 5C).[32,33] Altogether, the

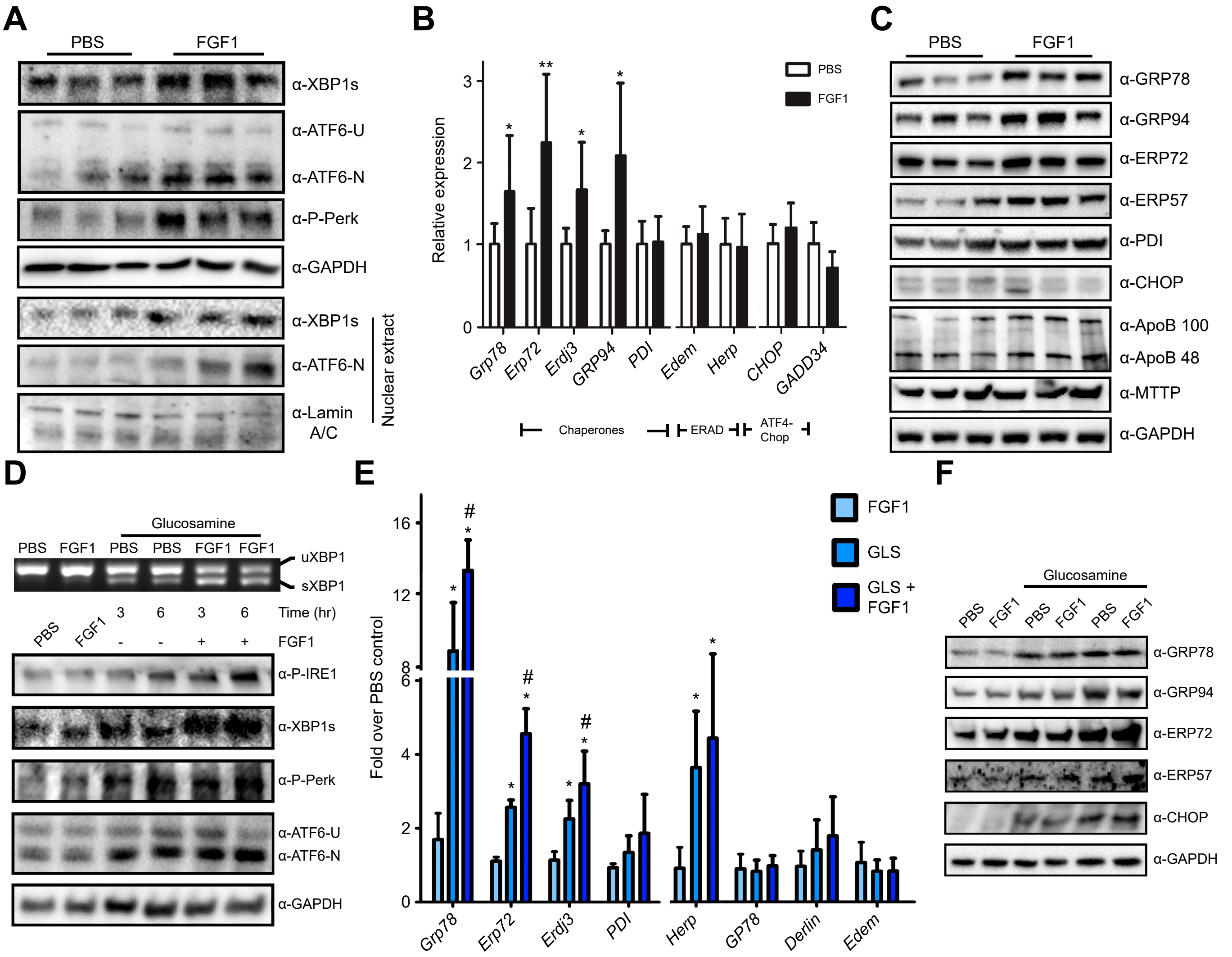

Fig. 4. FGF1 hyperactivates the UPR in the liver and hepatocytes. (A) Hepatic protein levels of the three UPR branch markers XBP1s, ATF6 (ATF6-U: unspliced & ATF6-N nuclear) and Perk phosphorylation 14 h after FGF1 injection of *ob/ob* showing UPR activation. In addition, XBP1s and ATF6 in nuclear extracts from these livers. (B) Hepatic expression levels of UPR chaperones (*Grp78*, *Erp72*, *Erdj3*, *Grp94*, and *Pdi*), ER-associated protein degradation genes (*Edem* and *Herp*), and PERK–ATF4–CHOP pathway genes (*Chop* and *Gadd34*) in ob/ob mouse liver (Mann-Whitney *U* test; **p* <0.05, ***p* <0.01). (C) Hepatic protein levels of Grp78, Grp94, Erp72, Erp57, Pdi and Chop 14 h after FGF1 injection of *ob/ob* mice. (D) Effect of 3 or 6 h FGF1 on UPR sensors in glucosamine-stressed HepG2 cells, showing *Xbp1* mRNA (uXBP1: unspliced & sXBP: spliced), Ire1 and Perk phosphorylation, XBP1s and Atf6 protein levels (unspliced & nuclear) (E) Hyperactivation of UPR chaperone expression levels but not ERAD genes by 6 h FGF1 treatment in glucosamine-stressed (2 h) primary rat hepatocytes. **p* <0.05 glucosamine *vs.* control, #*p* <0.05 glucosamine *vs.* glucosamine + FGF1 (Kruskal-Wallis test). (F) UPR chaperone protein induction after FGF1 treatment in HepG2 cells with and without glucosamine pre-treatment. UPR, unfolded protein response.

hyperactivation of the UPR by FGF1 depends on the regulatory signaling cascades controlling protein synthesis and ER protein folding capacity in hepatocytes.

In vivo modulation of ER stress affects the TG-promoting activity of FGF1

To establish whether the VLDL-promoting effect of FGF1 also depends on pre-existing ER stress *in vivo,* we tested whether modulation of ER stress interfered with the FGF1-dependent increase in plasma TG levels. While FGF1 had a modest effect on plasma TG levels in lean mice, a condition with minimal ER stress in the liver (Fig. 6A), FGF1 adopted novel TG secretory activity in ER stress-primed lean mice using a low dose of tunicamycin (Fig. 6B). This dose of tunicamycin was ten times lower than a previously described sublethal dose and did not lead to liver damage as indicated by unchanged levels of plasma ALT (Fig. 6C). As the presence of ER stress in an otherwise healthy liver provided the conditions that enable FGF1-induced TG secretion, we next tested whether a reduction of ER stress in steatotic livers had the opposite effect. Indeed, a 3-day pre-treatment of DIO mice with the ER chaperone phenylbutyrate that decreased plasma ALT levels (Fig. 6D), also abrogated the FGF1-stimulated increase in plasma TG levels (Fig. 6E). Similar to *ob/ob* mice, DIO mice, which represent a more relevant model for obesity-driven metabolic disease, also displayed increased hepatic protein levels of Grp78 as well as ApoB after treatment with FGF1

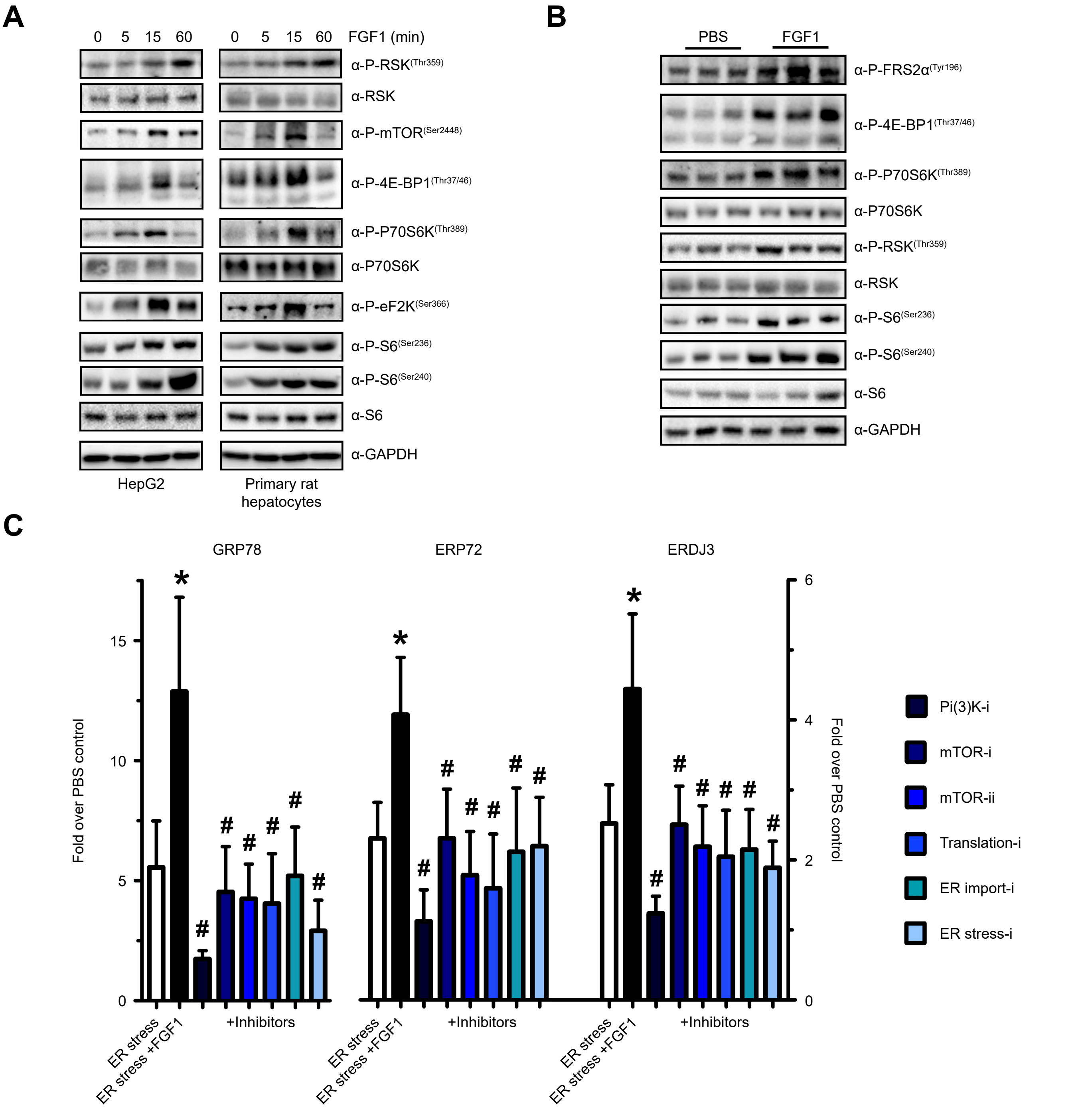

Fig. 5. FGF1 stimulates major regulators of protein synthesis that link to the UPR. (A) Temporal phosphorylation dynamics of signaling pathways in FGF1-stimulated HepG2 cells and primary rat hepatocytes: RSK, mTOR and downstream targets, 4E-BP1, P70S6K, eF2K and S6. (B) Phosphorylation of FRS2α, 4E-BP1, P70S6K, RSK, and S6 in livers of DIO mice 15 min after FGF1 stimulation. (C) Suppression of FGF1-induced UPR hyperactivation by inhibition of PI3K, mTOR, protein translation, ER protein import and a chemical protein folding chaperone in HepG2 cells (Kruskal-Wallis-test). *p <0.05 ER stress *vs.* ER stress + FGF1, $^{\#}p$ <0.05 ER stress + FGF1 *vs.* ER stress + FGF1 + inhibitor. DIO, diet-induced obese; ER, endoplasmic reticulum; UPR, unfolded protein response.

(Fig. S6). Consistent with a generic synthetic mechanism for VLDL-TG secretion, such as a canonical UPR, the most abundant plasma protein – albumin, produced by hepatocytes – was also induced by FGF1 in *ob/ob* but not lean mice (Fig. 6F). Finally, plasma ALT levels showed a transient reduction in response to FGF1 treatment, suggesting that FGF1-stimulated UPR activation is adaptive and hepatoprotective (Fig. 6G).

Together, our findings reveal a mechanism in which pharmacologically administered FGF1 acutely stimulates an adaptive UPR, leading to increased ER secretory capacity under pathophysiological (*e.g.* MASLD) conditions and a

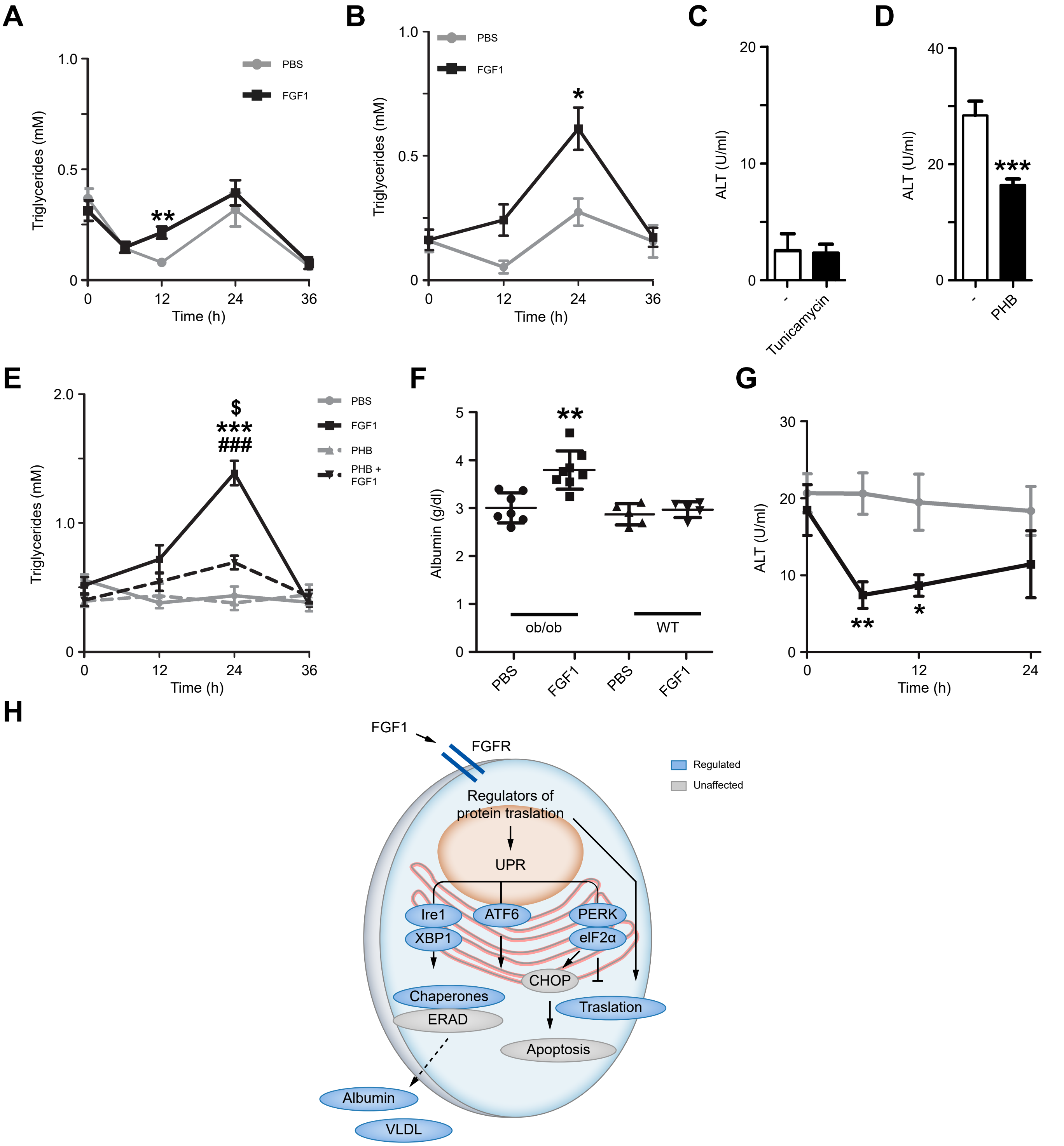

Fig. 6. FGF1-induced hepatic TG clearance requires pre-existing ER stress. Temporal dynamics of plasma TG upon an FGF1 injection in (A) wild-type mice, (B) wild-type mice pre-treated with tunicamycin (Mann-Whitney *U* test; $^*p$ <0.05, $^{**}p$ <0.01). (C) Plasma ALT levels after tunicamycin pre-treatment showing no liver damage and (D) after 3-days of phenylbutyrate pre-treatment in DIO mice showing ER liver damage reduction (Mann-Whitney *U* test; $^{***}p$ <0.001). (E) Temporal dynamics of plasma TG upon an FGF1 injection in DIO mice with or without pre-treatment for 3 days with the ER chaperone phenylbutyrate (Kruskal-Wallis-test; $^{***}p$ <0.001 FGF1 *vs.* PBS, $^{\#\#\#}p$ <0.001 FGF1 *vs.* PHB, $p <0.05 FGF1 *vs.* PHB+FGF1). (F) Albumin levels in *ob/ob* and wild-type mice 24 h after FGF1 injection (Mann-Whitney *U* test; $^{***}p$ <0.01). (G) Temporal dynamics of plasma ALT in *ob/ob* mice after a single FGF1 injection showing a transient reduction in liver damage (Mann-Whitney *U* test; $^*p$ <0.05, $^{**}p$ <0.01). (H) Model summarizing the underlying mechanism of FGF1-induced clearance of hepatic steatosis; in blue, the elements of the signaling pathways and UPR that are affected downstream of FGF1 binding and the subsequent secretory output. n = 5-6, n = 11 for panel E. $^*p$ <0.05, $^{**}p$ <0.01. ALT, alanine aminotransferase; DIO, diet-induced obese; ER, endoplasmic reticulum; TG, triglyceride; UPR, unfolded protein response.

transient increase in VLDL secretion, ultimately clearing hepatic steatosis (Fig. 6H).

Discussion

Previously, we and others have shown that FGF1 effectively reduces hepatic steatosis in mouse models of MASLD.[12,14,19] Here we show that the anti-steatotic properties of FGF1 can be explained by its ability to acutely stimulate the secretion of TG-containing VLDL particles through a transient adaptive UPR. This effect is independent of adipose FGFR1 and therefore independent of the known anti-lipolytic and glucose-lowering effects of FGF1, as well as the indirect effects of adipose FGFR1 on hepatic lipid metabolism.[17,34] Although increased hepatic clearance of TGs is favorable for the liver, chronically elevated plasma TG-rich lipoproteins predispose to cardiovascular disease via uptake of their remnants into the arterial wall. However, chronic FGF1 treatment was not found to be linked to hyperlipidemia, suggesting adequate peripheral clearance of TG-rich particles.[12] FGF1-induced hepatic TG secretion was accompanied by increased whole-body lipid utilization. Whether this shift toward lipids as fuel is primarily driven by hepatic lipid secretion, by the limited availability of carbohydrates due to the acute glucose-lowering effects of FGF1, or by the described reduction in food intake warrants further investigation. As the glucose-lowering effects of FGF1 have been attributed to direct actions on both adipose tissue and muscle, it is conceivable that these tissues could be responsible for the VLDL-derived fatty acid clearance.[17,35]

Despite the beneficial hepatic effect of FGF1, ApoB lipoprotein particles (especially LDL-cholesterol) are central to atherosclerosis development and even with effective LDL-cholesterol control, residual risk is prevalent in patients with metabolic syndrome. Therefore, potential cardiovascular consequences need to be thoroughly addressed before considering FGF1 as a therapeutic target for metabolic disease. MASLD is also an independent risk factor for cardiovascular disease that intertwines with insulin resistance, another strong predictor of cardiovascular events that is also positively influenced by FGF1.[12,36] The same holds for inflammation and blood pressure, and the link between FGF1 and cardiovascular outcomes is multifactorial.[37,38]

In addition to transient VLDL stimulation, FGF1 also altered the levels of other plasma proteins of hepatic origin such as albumin, ALT, and coagulation factors. FGF1 might therefore have a broad effect on the synthetic function of the liver. Accordingly, a canonical UPR was identified as the underlying mechanism. Although we cannot rule out that the induction of a specific UPR component is responsible for a direct effect on VLDL secretion, collaborative action of all three UPR branches is needed to maintain ER protein processing and lipid homeostasis.[39,40] Instead, a disruption of the temporal dynamics by the chronic unresolved conditions that characterize MASLD has been proposed to underlie the shift toward a derailed maladaptive UPR with metabolic consequences.[41] As FGF1 also impinges on these temporal dynamics, we aimed to evaluate the impact of FGF1 on VLDL secretion by modulating ER stress in an acute setting rather than with genetic models, showing that ER stress is both sufficient and required for stimulation of VLDL production by FGF1. After prolonged treatment, a plasma TG response similar to that seen after a single FGF1 administration was observed, indicating that resistance to the treatment does not develop. Even longer treatment regimens did not identify a reduction in potency.[12,19] Yet, only a minor response was observed in lean mice, suggesting that the response is proportional to the extent of hepatic lipid accumulation and the accompanying ER stress. As the anti-steatotic property of FGF1 can be uncoupled from its mitogenic characteristics, the major drawback of applying non-modified FGF1 as a therapeutic can be circumvented.

FGF1 could affect the UPR pathway in models of obesity and MASLD, even after the chronic ER stress conditions that dampen the potential of the pathway had manifested in the liver. These finding imply that regardless of the conditions, it remains possible to trigger a transition from a deleterious to an adaptive UPR.

Protein secretion is one of the most demanding processes in the liver, and approximately 40% of hepatic proteins are secreted.[42] Therefore, secretory function must be highly integrated with cellular homeostasis. We showed that the major regulators of protein synthesis such as mTORC1 are required for UPR activation by FGF1 in hepatocytes and are also activated in mouse liver. The ability to modulate hepatic protein synthesis was also shown for FGF19,[29] yet only by ERK-P90RSK, whereas FGF1 also activates the PI3K-Akt-mTORC1 pathway. The master regulator of protein synthesis, mTORC1 is intimately connected to the UPR.[43,44] Paradoxically, both of these pathways have been linked to exacerbation as well as amelioration of MASLD by impinging on various metabolic lipid pathways.[45–48] However, other than VLDL production, no other lipid fluxes were affected by FGF1. The complex context of MASLD, which is characterized by insulin resistance, lipotoxicity and oxidative as well as ER stress may explain why some but not all of the pathways that can potentially be modulated by mTORC and the UPR, are acutely activated by FGF1.[49]

Next to protein synthesis, FGF19 directly suppresses the rate-limiting enzyme in hepatic bile acid synthesis, *Cyp7A1,* in an FGFR4-dependent manner. We show that FGF1 also suppresses Cyp7A1 and activates FGF receptor substrate in the liver, as well as P90RSK in a similar fashion to FGF19.[29] The anti-steatotic effects of the universal ligand FGF1 also appear to rely on direct hepatic signaling through FGF receptors, likely FGFR4, yet without dependency on cofactors such as β-klotho[19,50] Whether the positive effects of FGF19 analogs on MASLD and MASH in recent trials also hold true for FGF1 and whether the mechanism identified here is also relevant for FGF19 and its analogs, remains to be investigated.[50]

In conclusion, we show that the anti-steatotic action of systemically administered FGF1 is driven by a transient UPR-dependent modulation of hepatic VLDL secretion. FGF1 signaling ties into a pathophysiological state of pre-existing chronic ER stress, ultimately clearing hepatic steatosis. Our findings provide a rationale for the potent anti-steatotic action of FGF mimetics, thereby offering novel strategies for the prevention or treatment of MASLD.

Affiliations

[1]*Department of Pediatrics, University of Groningen, University Medical Center Groningen, Groningen, The Netherlands;* [2]*Faculty Campus Fryslân, University of Groningen, The Netherlands;* [3]*Gene Expression Laboratory, Salk Institute for Biological Studies, 10010 North Torrey Pines Road, La Jolla, California 92037, USA*

Abbreviations

ALT, alanine aminotransferase; Apo, apolipoprotein; DIO, diet-induced obese; ER, endoplasmic reticulum; Fatp, fatty acid transport protein; FGF, fibroblast growth factor; FGFR, fibroblast growth factor receptor; FPLC, fast protein liquid chromatography; LDL, low-density lipoprotein; MASLD, metabolic dysfunction-associated steatotic liver disease; MASH, metabolic dysfunction-associated steatohepatitis; P70S6K, P70 ribosomal S6 kinases; P90RSK, P90 ribosomal S6 kinases; TG, triglyceride; VLDL, very low-density lipoprotein; UPR, unfolded protein response; Xbp1s, X-box binding protein 1s.

Financial support

This study was supported by grants from The Netherlands Organization for Scientific Research (VICI grant 016.176.640 to JWJ), the De Cock Stichting, and the European Foundation for the Study of Diabetes (award supported by EFSD/ Novo Nordisk). This work was further supported by grants to RME from the National Institutes of Health through the National Institute of Diabetes and Digestive and Kidney Diseases (DK057978 and DK120515) and the National Heart, Lung, and Blood Institute (HL147835) as well as the Larry L. Hillblom Foundation, Inc. (2021-D-001-NET). The content is solely the responsibility of the authors and does not necessarily represent the official views of the NIH. RME holds the March of Dimes Chair in Molecular and Developmental Biology at the Salk Institute and is a previous Nomis Distinguished Scholar.

Conflict of interest

The authors declare no conflicts of interest.
Please refer to the accompanying ICMJE disclosure forms for further details.

Authors' contributions

DS, TvZ, and JWJ designed and supervised the research. TvZ, DS, WL, SL and JCW performed experiments and procedures. TvZ, DS, WL, SL, BPS performed formal analysis. TvZ, DS and JWJ wrote the original draft of the manuscript. All authors contributed to review and editing of the final manuscript.

Data availability

The data used to support the findings of this study, if not found in the manuscript and its supporting information files, are available from the corresponding author upon reasonable request.

Acknowledgements

The authors would like to thank Theo van Dijk and Ydwine van der Veen (Department of Pediatrics, University Medical Center Groningen) for technical assistance.

Supplementary data

Supplementary data to this article can be found online at https://doi.org/10.1016/j.jhepr.2025.101660.

References

Author names in bold designate shared co-first authorship

[1] Eslam M, Sanyal AJ, George J. International consensus panel MAFLD: a consensus-driven proposed nomenclature for metabolic associated fatty liver disease. Gastroenterology 2020;158:1999–2014.

[2] Younossi ZM, Wong G, Anstee QM, et al. The global burden of liver disease. Clin Gastroenterol Hepatol 2023;21:1978–1991.

[3] Cai J, Zhang XJ, Ji YX, et al. Nonalcoholic fatty liver disease pandemic fuels the upsurge in cardiovascular diseases. Circ Res 2020;126:679–704.

[4] Byrne CD, Targher G. NAFLD a multisystem disease. J Hepatol 2015;62: S47–S64.

[5] **Liu SS**, **Ma XF**, Zhao J, et al. Association between nonalcoholic fatty liver disease and extrahepatic cancers: a systematic review and meta-analysis. Lipids Health Dis 2020;19:118.

[6] Ajoolabady A, Kaplowitz N, Lebeaupin C, et al. Endoplasmic reticulum stress in liver diseases. Hepatology 2023;77:619–639.

[7] Koullias E, Papavdi M, Koskinas J, et al. Targeting metabolic dysfunction-associated steatotic liver disease (MASLD): available and future pharmaceutical options. Cureus 2025;17(1):e76716.

[8] Sanyal A, Charles ED, Neuschwander-Tetri BA, et al. Pegbelfermin (BMS-986036), a PEGylated fibroblast growth factor 21 analogue, in patients with non-alcoholic steatohepatitis: a randomised, double-blind, placebo-controlled, phase 2a trial. Lancet 2019;392:2705–2717.

[9] Struik D, Dommerholt MB, Jonker JW. Fibroblast growth factors in control of lipid metabolism : from biological function to clinical application. Curr Opin Lipidol 2019;3:235–243.

[10] **Tian H**, **Zhang S**, Liu Y, et al. Fibroblast growth factors for nonalcoholic fatty liver disease: opportunities and challenges. Int J Mol Sci 2023;24:4583.

[11] **Jonker JW**, **Suh JM**, Atkins AR, et al. A PPARγ-FGF1 axis is required for adaptive adipose remodelling and metabolic homeostasis. Nature 2012;485:391–394.

[12] **Suh JM**, **Jonker JW**, Ahmadian M, et al. Endocrinization of FGF1 produces a neomorphic and potent insulin sensitizer. Nature 2014;513:436–439.

[13] **Huang Z**, **Tan Y**, Gu J, et al. Uncoupling the mitogenic and metabolic functions of FGF1 by tuning FGF1-FGF receptor dimer stability. Cell Rep 2017;20:1717–1728.

[14] Liu W, Struik D, Nies VJ, et al. Effective treatment of steatosis and steatohepatitis by fibroblast growth factor 1 in mouse models of nonalcoholic fatty liver disease. Proc Natl Acad Sci USA 2016;113:2288–2293.

[15] Fu T, Choi SE, Kim DH, et al. Aberrantly elevated microRNA-34a in obesity attenuates hepatic responses to FGF19 by targeting a membrane coreceptor β-Klotho. Proc Natl Acad Sci USA 2012;109:16137–16142.

[16] Aaldijk AS, Verzijl CRC, Jonker JW, et al. Biological and pharmacological functions of the FGF19- and FGF21-coreceptor beta klotho. Front Endocrinol (Lausanne) 2023;14:1150222.

[17] Sancar G, Liu S, Gasser E, et al. FGF1 and insulin control lipolysis by convergent pathways. Cell Metab 2022;34:171–183.

[18] **Xu Z**, **Wu Y**, **Wang F**, et al. Fibroblast growth factor 1 ameliorates diabetes-induced liver injury by reducing cellular stress and restoring autophagy. Front Pharmacol 2020;11:52.

[19] Lin Q, Huang Z, Cai G, et al. Activating adenosine monophosphate-activated protein kinase mediates fibroblast growth factor 1 protection from nonalcoholic fatty liver disease in mice. Hepatology 2021;73:2206–2222.

[20] Wegrzyn AB, Herzog K, Gerding A, et al. Fibroblast-specific genome-scale modelling predicts an imbalance in amino acid metabolism in Refsum disease. FEBS J 2020;287:5096–5113.

[21] Wijers M, Zanoni P, Liv N, et al. The hepatic WASH complex is required for efficient plasma LDL and HDL cholesterol clearance. JCI Insight 2019;4: e126462.

[22] Cox J, Hein MY, Luber CA, et al. Accurate proteome-wide label-free quantification by delayed normalization and maximal peptide ratio extraction, termed MaxLFQ. Mol Cell Proteomics 2014;13:2513–2526.

[23] Tyanova S, Temu T, Sinitcyn P, et al. The Perseus computational platform for comprehensive analysis of (prote)omics data. Nat Methods 2016;13:731–740.

[24] Bligh EG, Dyer WJ. A rapid method of total lipid extraction and purification. Can J Biochem Physiol 1959;37:911–917.

[25] Hirschey MD, Shimazu T, Goetzman E, et al. SIRT3 regulates mitochondrial fatty-acid oxidation by reversible enzyme deacetylation. Nature 2010;464: 121–125.

[26] Oosterveer MH, van Dijk TH, Tietge UJ, et al. High fat feeding induces hepatic fatty acid elongation in mice. PLoS One 2009;4:e6066.

[27] Moshage H, Casini A, Lieber CS. Acetaldehyde selectively stimulates collagen production in cultured rat liver fat-storing cells but not in hepatocytes. Hepatology 1990;12:511–518.

[28] Sniderman AD, Thanassoulis G, Glavinovic T, et al. Apolipoprotein B particles and cardiovascular disease: a narrative review. JAMA Cardiol 2019;4:1287–1295.

[29] Kir S, Beddow SA, Samuel VT, et al. FGF19 as a postprandial, insulin-independent activator of hepatic protein and glycogen synthesis. Science 2011;331:1621–1624.
[30] Rusli F, Deelen J, Andriyani E, et al. Fibroblast growth factor 21 reflects liver fat accumulation and dysregulation of signalling pathways in the liver of C57BL/6J mice. Sci Rep 2016;6:30484.
[31] Ruoff R, Katsara O, Kolupaeva V. Cell type-specific control of protein synthesis and proliferation by FGF-dependent signaling to the translation repressor 4E-BP. Proc Natl Acad Sci USA 2016;113:7545–7550.
[32] Özcan U, Yilmaz E, Ozcan L, et al. Chemical chaperones reduce ER stress and restore glucose homeostasis in a mouse model of type 2 diabetes. Science 2006;313:1137–1140.
[33] Xiao C, Giacca A, Lewis GF. Sodium phenylbutyrate, a drug with known capacity to reduce endoplasmic reticulum stress, partially alleviates lipid-induced insulin resistance and beta-cell dysfunction in humans. Diabetes 2011;60:918–924.
[34] Yang C, Wang C, Ye M, et al. Control of lipid metabolism by adipocyte FGFR1-mediated adipohepatic communication during hepatic stress. Nutr Metab (Lond) 2012;9(1):94.
[35] Ying L, Wang L, Guo K, et al. Paracrine FGFs target skeletal muscle to exert potent anti-hyperglycemic effects. Nat Commun 2021;12(1):7256.
[36] Linero PL, Castilla-Guerra L. Management of cardiovascular risk in the non-alcoholic fatty liver disease setting. Eur Cardiol 2024;19:e02.
[37] Cuevas P, Carceller F, Ortega S, et al. Hypotensive activity of fibroblast growth factor. Science 1991;254:1208–1210.
[38] Tomaszewski M, Charchar FJ, Lynch MD, et al. Fibroblast growth factor 1 gene and hypertension: from the quantitative trait locus to positional analysis. Circulation 2007;116:1915–1924.
[39] Wang S, Chen Z, Lam V, et al. IRE1α-XBP1s induces PDI expression to increase MTP activity for hepatic VLDL assembly and lipid homeostasis. Cell Metab 2012;16:473–486.
[40] **Rutkowski DT**, **Wu J**, Back SH, et al. UPR pathways combine to prevent hepatic steatosis caused by ER stress-mediated suppression of transcriptional master regulators. Dev Cell 2008;15:829–840.
[41] Fu S, Watkins SM, Hotamisligil GS. The role of endoplasmic reticulum in hepatic lipid homeostasis and stress signaling. Cell Metab 2012;15:623–634 (2012b).
[42] Preidis GA, Kim KH, Moore DD. Nutrient-sensing nuclear receptors PPARα and FXR control liver energy balance. J Clin Invest 2017;127:1193–1201.
[43] Appenzeller-Herzog C, Hall MN. Bidirectional crosstalk between endoplasmic reticulum stress and mTOR signaling. Trends Cell Biol 2012;22:274–282.
[44] Gaudette BT, Jones DD, Bortnick A, et al. mTORC1 coordinates an immediate unfolded protein response-related transcriptome in activated B cells preceding antibody secretion. Nat Commun 2020;11:723.
[45] Lee AH, Scapa EF, Cohen DE, et al. eRegulation of hepatic lipogenesis by the transcription factor XBP1. Science 2008;320:1492–1496.
[46] Peterson TR, Sengupta SS, Harris TE, et al. mTOR complex 1 regulates lipin 1 localization to control the SREBP pathway. Cell 2011;146:408–420.
[47] Kenerson HL, Subramanian S, McIntyre R, et al. Livers with constitutive mTORC1 activity resist steatosis independent of feedback suppression of Akt. PLoS One 2015;10:e0117000.
[48] Ashraf S, Ashraf N, Yilmaz G, et al. Crosstalk between beta-adrenergic and insulin signaling mediates mechanistic target of rapamycin hyperactivation in liver of high-fat diet-fed male mice. Physiol Rep 2021;9:e14958.
[49] Rutkowski DT. Liver function and dysfunction - a unique window into the physiological reach of ER stress and the unfolded protein response. FEBS J 2019;286:356–378.
[50] **Lin H**, **Zhou HC**, **Hou Y**, et al. Paracrine fibroblast growth factor 1 functions as potent therapeutic agent for intrahepatic cholestasis by downregulating synthesis of bile acid. Front Pharmacol 2019;10:1515.

Keywords: Fibroblast growth factor 1; Unfolded Protein Response; ER stress; VLDL secretion.
Received 1 April 2025; received in revised form 17 October 2025; accepted 23 October 2025; Available online 30 October 2025

Journal of Hepatology, Volume *8*

Supplemental information

FGF1 ameliorates hepatic steatosis through acute activation of the unfolded protein response and VLDL production

Tim van Zutphen, Dicky Struik, Weilin Liu, Sihao Liu, Benan Pelin Sermikli, Justina C. Wolters, Henkjan J. Verkade, Annette R. Atkins, Michael Downes, Ronald M. Evans, and Johan W. Jonker

FGF1 ameliorates hepatic steatosis through acute activation of the unfolded protein response and VLDL production

Tim van Zutphen, Dicky Struik, Weilin Liu Sihao Liu, Benan Pelin Sermikli, Justina C. Wolters, Henkjan J. Verkade, Annette R Atkins, Michael Downes, Ronald M. Evans, Johan W. Jonker

Table of contents

Fig. S1……………………………………………………………………………………2

Fig. S2……………………………………………………………………………………3

Fig. S3……………………………………………………………………………………5

Fig. S4……………………………………………………………………………………7

Fig. S5……………………………………………………………………………………8

Fig. S6……………………………………………………………………………………9

Table S1…………………………………………………………………………………..10

Table S2…………………………………………………………………………………..11

Fig. S1

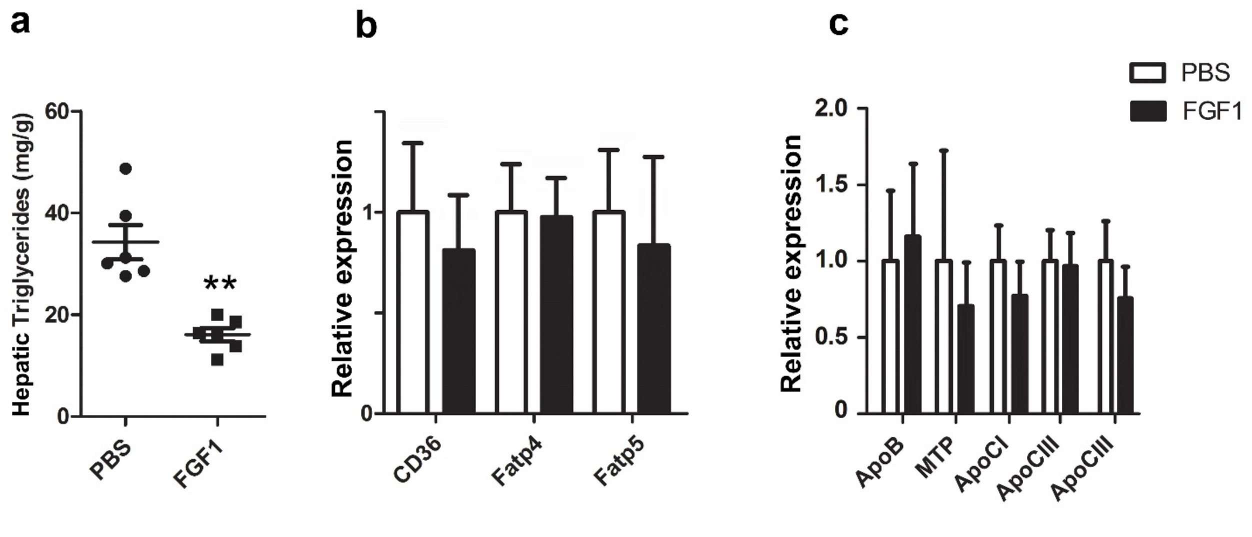

Fig. S1 (a) Hepatic triglyceride levels of *ob/ob* mice treated for 2 weeks with FGF1. (b) Hepatic expression levels of fatty acid transporter genes *Cd36*, *Fatp4* and *Fatp5* and (c) apolipoprotein genes 14-hours after FGF1 stimulation in *ob/ob*. All experiments n=6-8.

Fig. S2

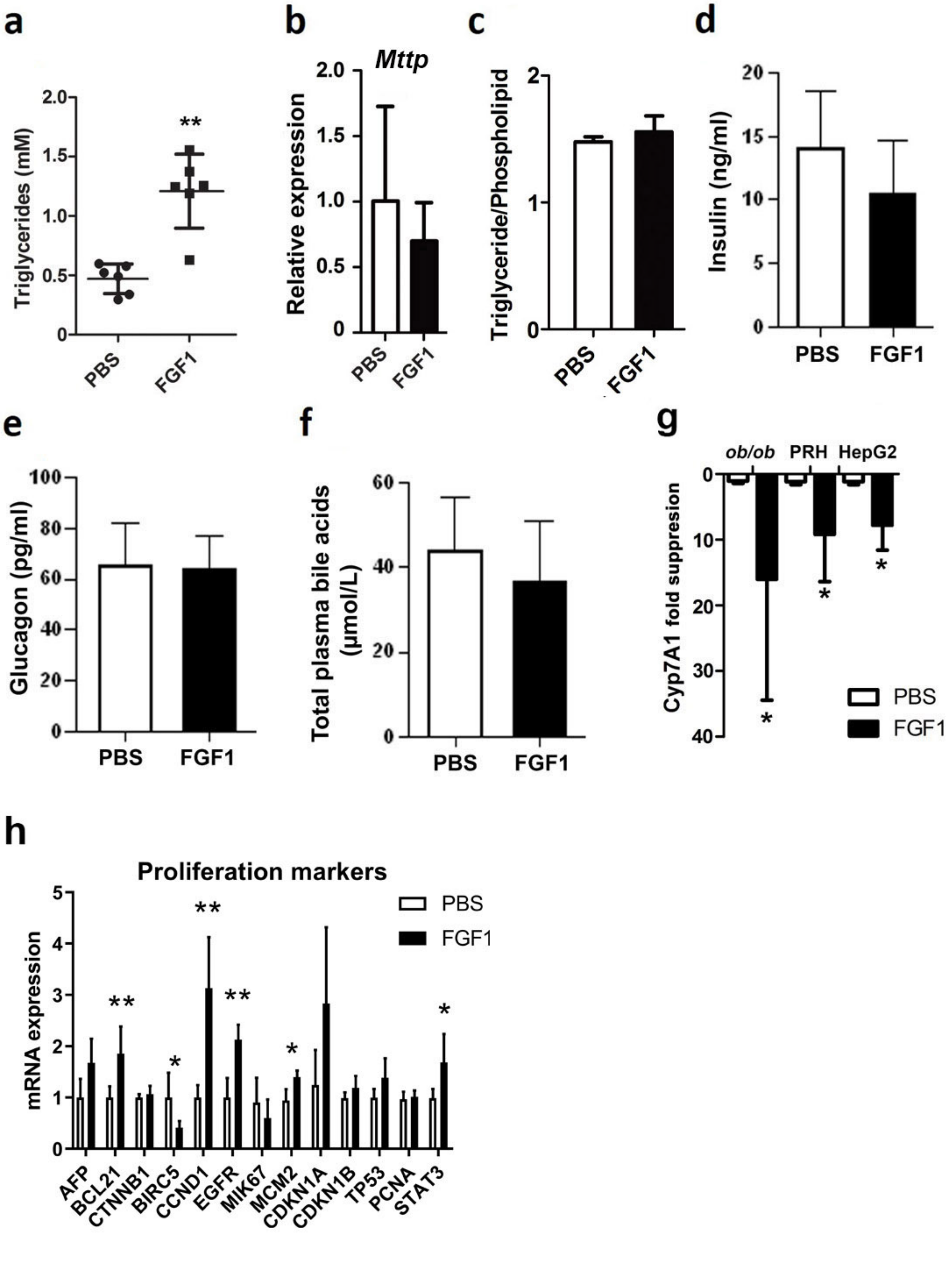
a
Triglycerides (mM)
**
PBS
FGF1
b
Mttp
Relative expression
PBS
FGF1
c
Triglyceride/Phospholipid
PBS
FGF1
d
Insulin (ng/ml)
PBS
FGF1
e
Glucagon (pg/ml)
PBS
FGF1
f
Total plasma bile acids (μmol/L)
PBS
FGF1
g
ob/ob
PRH
HepG2
Cyp7A1 fold suppresion
PBS
FGF1
h
Proliferation markers
mRNA expression
PBS
FGF1
AFP
BCL21
CTNNB1
BIRC5
CCND1
EGFR
MIK67
MCM2
CDKN1A
CDKN1B
TP53
PCNA
STAT3

Fig. S2. (a) Plasma triglycerides of *ob/ob* 24-hours after the last FGF1 administration of a 2-week treatment (Mann-Whitney U-test), **p<0,01. (b) Hepatic expression levels of microsomal triglyceride transfer protein (*Mttp*) in *ob/ob* 14 hours after an FGF1 injection. (c) Plasma insulin (d), plasma glucagon (e), and total bile acid concentrations (f) 24 hours after injection in *ob/ob.* (g) Suppression of bile acid synthesis gene *Cyp7A1* expression in *ob/ob* liver, primary rat hepatocytes and HepG2 by FGF1 (after 14, 16 & 16 hours respectively, Mann-Whitney U-test), *p<0,05. (h) Proliferation marker gene expression in *ob/ob* liver 6 hours after FGF1 injection. All experiments n=6-8.

Fig. S3

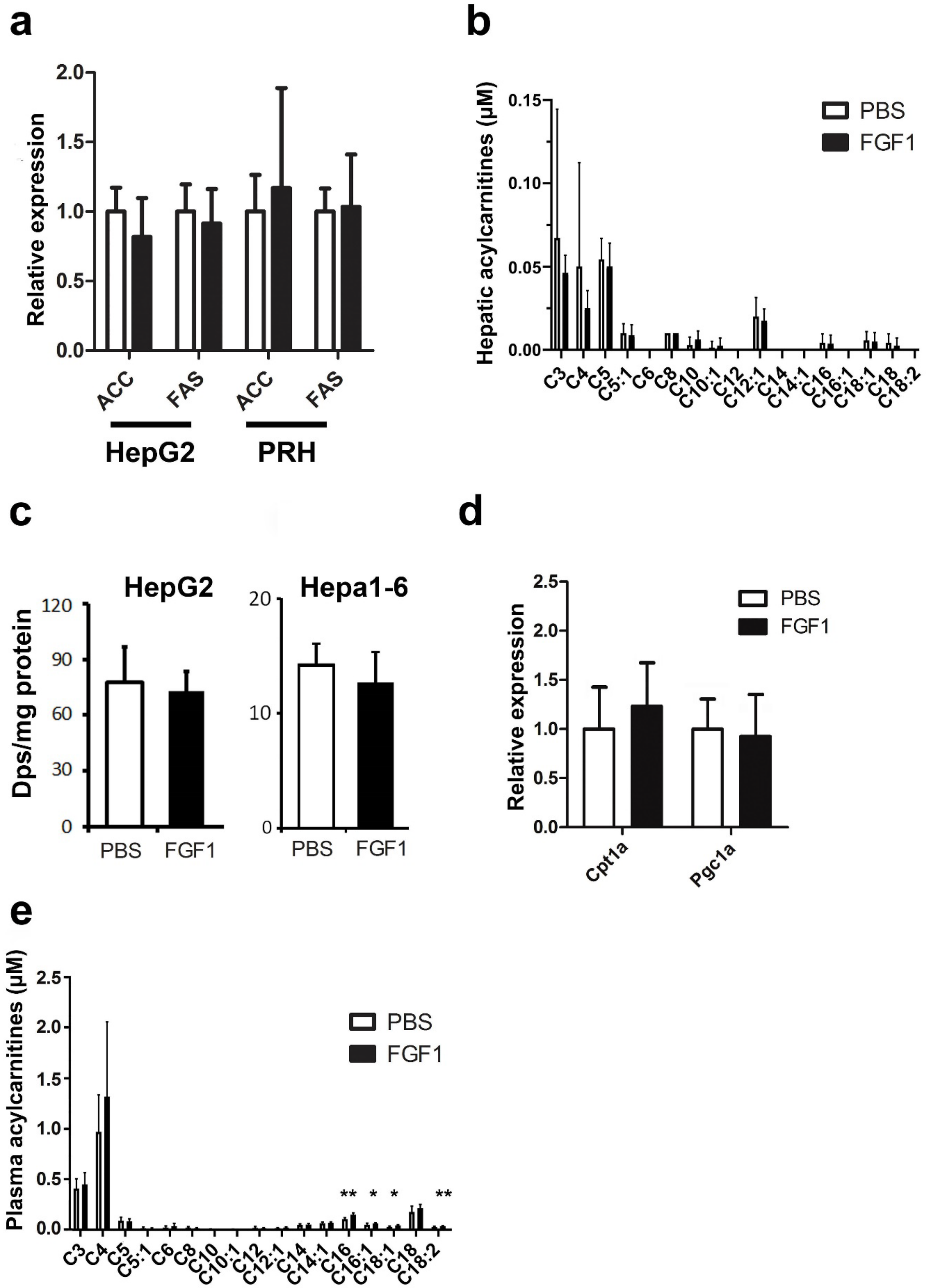
a
Relative expression
2.0
1.5
1.0
0.5
0.0
ACC
FAS
ACC
FAS
HepG2
PRH
b
Hepatic acylcarnitines (µM)
0.15
0.10
0.05
0.00
PBS
FGF1
C3
C4
C5
C5:1
C6
C8
C10
C10:1
C12
C12:1
C14
C14:1
C16
C16:1
C18:1
C18
C18:2
c
Dps/mg protein
HepG2
120
90
60
30
0
PBS
FGF1
Hepa1-6
20
10
0
PBS
FGF1
d
Relative expression
2.5
2.0
1.5
1.0
0.5
0.0
PBS
FGF1
Cpt1a
Pgc1a
e
Plasma acylcarnitines (µM)
2.5
2.0
1.5
1.0
0.5
0.0
PBS
FGF1
**
*
*
**
C3
C4
C5
C5:1
C6
C8
C10
C10:1
C12
C12:1
C14
C14:1
C16
C16:1
C18:1
C18
C18:2

Fig. S3. (a) Expression levels of lipogenesis genes Acetyl-CoA carboxylase (*Acc*) and Fatty acid synthase (*Fas*) in 6h FGF1-stimulated HepG2 cells and primary rat hepatocytes. (b) Acyl carnitines in liver of 14h FGF1-treated *ob/ob* mice. (c) β-oxidation rates in isolated mitochondria from HepG2 and Hepa1-6 cells after 6h FGF1 administration. (d) Hepatic expression levels of mitochondrial regulators *Cpt1a* and *Pgc1α* in 14h treated *ob/ob* mice. (e) Acyl carnitines in plasma of 14h FGF1-treated *ob/ob* mice (Mann-Whitney U-test), *p<0,05, **p<0,01. All experiments n=6-8.

Fig. S4

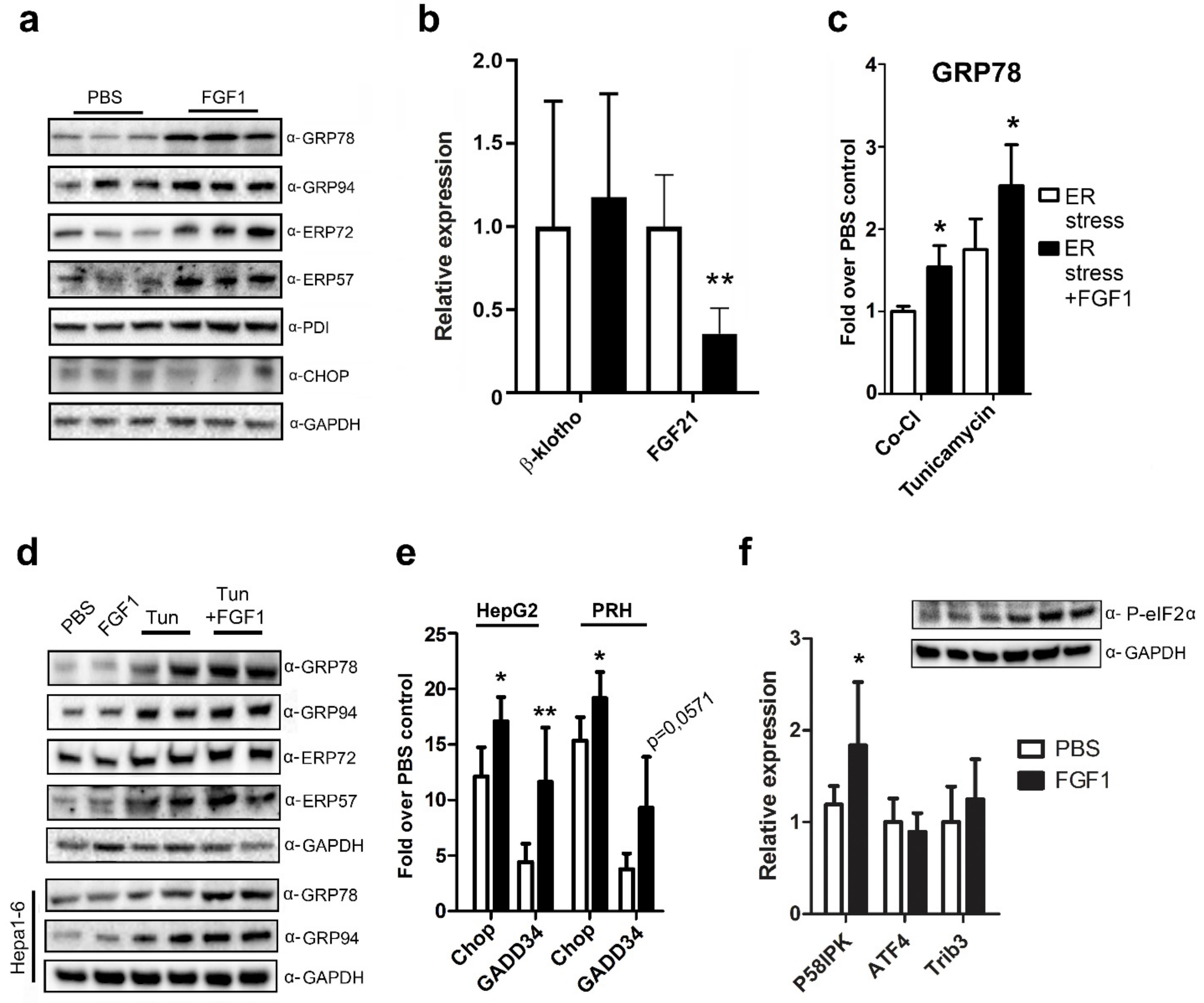

Fig. S4 (a) Hepatic UPR chaperone protein levels 24-hrs after last injection of a two-week FGF1 treatment in *ob/ob* mice. (b) Expression of *β-klotho* and *Fgf21* in same livers. (c) *Grp78 h*yperactivation in HepG2 by FGF1 upon 3-hour pretreatment with stressors such as cobalt and tunicamycin (Mann-Whitney U-test), *p<0,05. (d) Hyperactivation in HepG2 of UPR chaperone protein levels of Grp78, Grp94, Erp72 and Erp57 as well as in Hepa1-6 cells upon pretreatment with tunicamycin. (e) Hyperactivation of Perk-Atf4-Chop pathway target genes *Chop* and *Gadd34* by FGF1 in HepG2 and primary rat hepatocytes upon glucosamine pre-treatment (that is not observed *in vivo* in Figure 4A,B or Fig S4F, Mann-Whitney U-test), *p<0,05, **p<0,01. (f) Phosphorylation of eIF2α, but not induction of downstream targets *ATF4* and *Trib3* in livers of 14h FGF1-treated *ob/ob* mice (Mann-Whitney U-test), *p<0,05. All experiments n=6-8.

Fig. S5

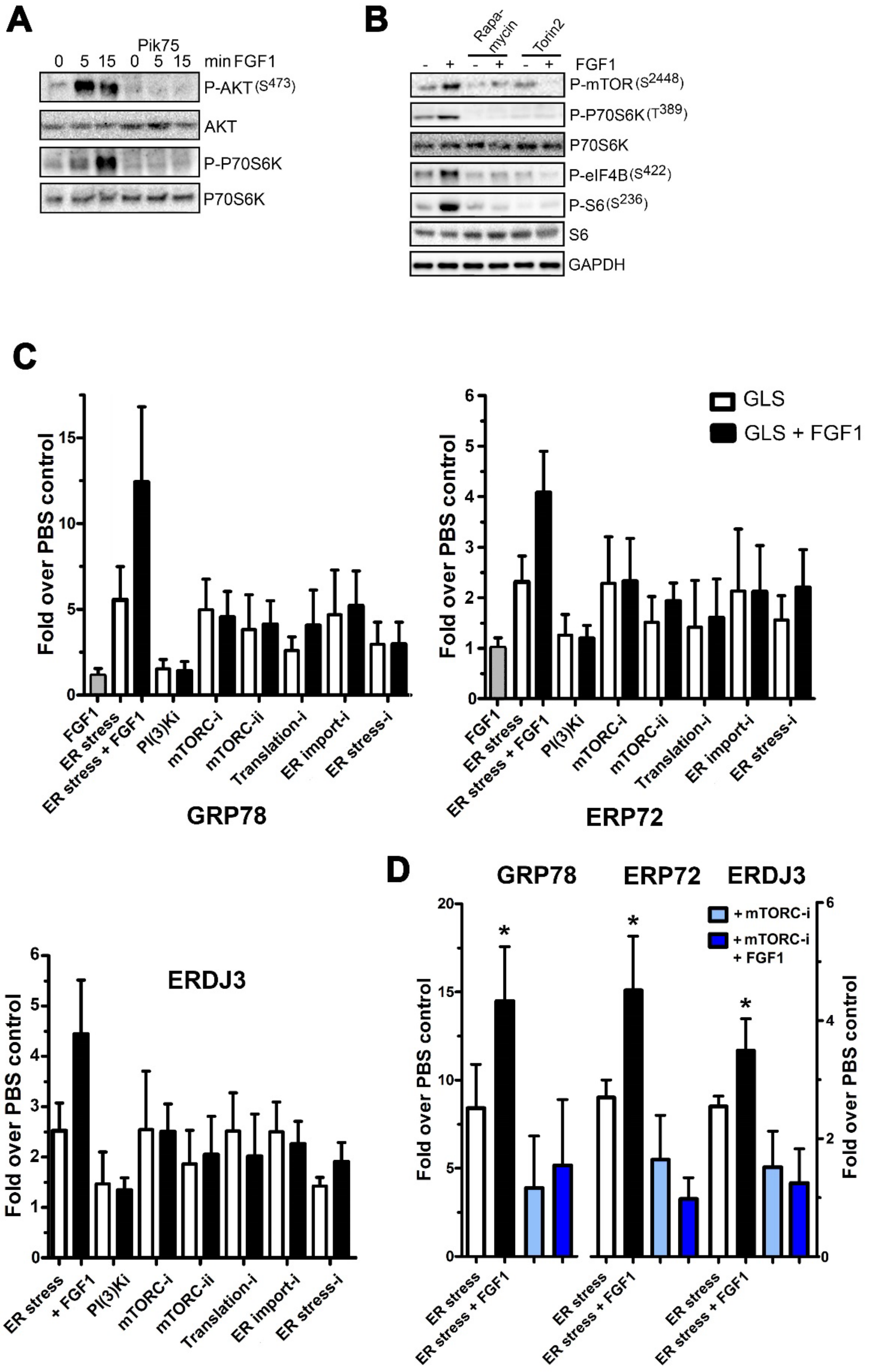
A
Pik75
0 5 15 0 5 15 min FGF1
P-AKT (S473)
AKT
P-P70S6K
P70S6K
B
Rapa-mycin
Torin2
- + - + - + FGF1
P-mTOR (S2448)
P-P70S6K (T389)
P70S6K
P-eIF4B (S422)
P-S6 (S236)
S6
GAPDH
C
GLS
GLS + FGF1
Fold over PBS control
FGF1
ER stress
ER stress + FGF1
PI(3)Ki
mTORC-i
mTORC-ii
Translation-i
ER import-i
ER stress-i
GRP78
ERP72
ERDJ3
D
GRP78
ERP72
ERDJ3
+ mTORC-i
+ mTORC-i + FGF1
ER stress
ER stress + FGF1

Fig. S5 (a) Suppressive effect of PI3K inhibition (by PIK-75) on 15 min FGF1-stimulated phosphorylation of AKT and P70S6K in HepG2 cells; (b) Suppressive effect of mTOR inhibition (by Rapamycin and Torin-2) on 15 min FGF1-stimulated phosphorylation of P70S6K, eIF4B and S6 in HepG2 cells. (c) Expanded representation of Figure 4D, suppression of hyperactivation of UPR chaperones *Grp78*, *Erp72* and *Erdj3*, including the controls that received the indicated inhibitors without the subsequent FGF1 stimulation. (d) Suppressive effect of mTOR inhibition (by Rapamycin and Torin-2) on hyperactivation of the UPR chaperones *Grp78*, *Grp94* and *Erdj3* in primary rat hepatocytes (Mann-Whitney U-test), *p<0,05, **p<0,01. All experiments n=6-12.

Fig. S6

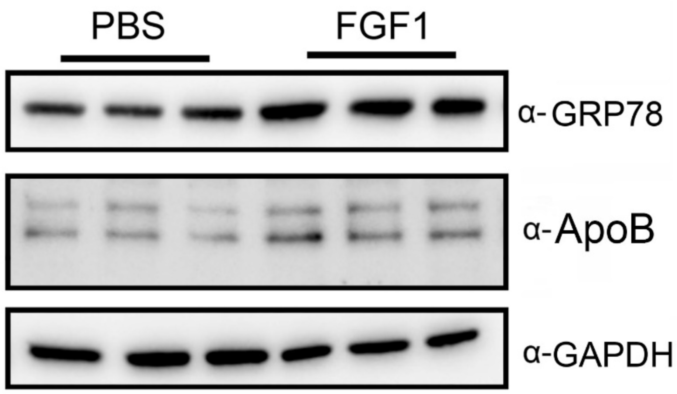

Fig. S6 Grp78 and ApoB protein levels in livers of diet-induced obese mice treated with FGF1 for 14 hours.

Table S1

Gene	Forward 5'-3'	Reverse 5'-3'
hAcc	CTCATCCAAACAGAGGGAACATC	CATGGGTCATGCCATAGTGGT
m/rAcc	CCATCCAAACAGAGGGAACATC	CTACATGAGTCATGCCATAGTGGT T
mApoB	GCCCATTGTGGACAAGTTGATC	CCAGGACTTGGAGGTCTTGGA
mApoCI	GGGCAGCCATTGAACATATCA	TTGCCAAATGCCTCTGAGAAC
mApoCII	TTACTGGACCTCTGCCAAGGA	CCCTGAGTTTCTCATCCATGC
mApoCIII	CCAAGACGGTCCAGGATGC	ACTTGCTCCAGTAGCCTTTCAGG
mbKlotho	ACACAACCTGATCAAGGCACA	CCTTCTGATGAGGGCGGAAG
mCct	ACCTGGCCCTAATGGAGCA	TTGGAGCTGGCTGCCGTAAACC
mCD36	GATCGGAACTGTGGGCTCAT	GGTTCCTTCTTCAAGGACAACTTC
hChop	GGAAATGAAGAGGAAGAATCAAAAAT	GTTCTGGCTCCTCCTCAGTCA
mChop	CAGGAAACGAAGAGGAAGAATCA	GCTCCTCTGTCAGCCAAGCTA
rChop	CAGGAAACGAAGAGGAAGAATCA	TAGCTTGGCTGACTGAGG
mCptT1α	CTCAGTGGGAGCGACTCTTCA	GGCCTCTGTGGTACACGACAA
h/m/rDerlin	GGAGGCCAGCAGACTATTTATTC	-
h/rDerlin	GGAGGCCAGCAGACTATTTATTC	GGCCTTAAATCGTGTTCCAAACC
mDerlin	GGAGGCCAGCAGACTATTTATTC	CTTAAATCGCGTTCCGAACC
h/m/rEdem1	ATGAACACCTGGATTGACTC	-
h/mEdem1	ATGAACACCTGGATTGACTC	AGGCAGATGGCATCTTCCAC
rEdem1	ATGAACACCTGGATTGACTC	AGGCAGATGGCGTCTTCCAC
h/m/rErdj3	CGCTTCCAAATGACCCAGG	ATAAAGGGGTACTCCATGCC
hErp72	GATGTCTCTGGCTATCCCACC	GGACTGCTCGATCATGTAATC
mErp72	ACGCCACCGAACAGACAGAC	CCAGACTGCTCAATCATGTAG
rErp72	ACGCCACCGAACAGACAGAC	CCAGACTGCTCAACCATGTAG
mFatp4	CCAGACAAGGGTTTTACAGATAAGCT	ACCTGCTGTGCACCACAATG
mFatp5	GTGCTGATTGTGGATCCAGAC	GAATGTTCTCAGCTAGCAGCTTG
hFAS	GATGACATCGTCCATTCGTTTGT	CCATGCAGCTCAGCAGGTCTA
m/rFAS	GGCATCATTGGGCACTCCTT	GCTGCAAGCACAGCCTCTCT
mFGF21	ACGACCAAGACACTGAAGCC	TTGAGCTCCAGGAGACTTTCTG
hGadd34	GGCTCAAGCGCCCAGAAACC	AATGGACAGTGACCTTCTCG
mGadd34	CCCGAGATTCCTCTAAAAGC	CCAGACAGCAAGGAAATGG
rGadd34	ACCCTGAGATTCCTCTGAAGG	ATCTCGTGCAAACTGCTCC
hGRP78	TGGTGATCAAGATACAGGTGACCT	GTGTTCCTTGGAATCAGTTTGGT
mGRP78	TGGTGATCAGGATACAGGTGATCT	GGTACCACAGTGTTCCTTGGAA
rGRP78	TGGTGATCAAGATACAGGTGACCT	GGTGGGCACCACAGTGTTCC
h/m/rGrp94	CCTGAGAGAACTGATTTCAAATGC	TGTCTGTGACATGCAGCAGG
h/m/rGp78	TGGCTTTCATGGCTGCAGAG	TCCCTTCGTGGTTGAGGTCC
h/m/rHerp	CTCAAGGCCCACCTGAGCCG	CAGCTTCCCAGAATAAATTAACC
mLpcat3	GGCCTCTCAATTGCTTATTT	AGGATGAGGAACTGAAGCAC
mMttp	CAAGCTCACGTACTCCACTGAAG	TCATCATCACCATCAGGATTCCT
hPDI1	CAAAATCAAGCCCCACCTGAT	CTGTTTGCAGTGACCACACC
m/rPDI1	CAAGATCAAGCCCCACCTGAT	CTGCTTGCAGTGACCACACC
mPemt	AGGAGTCCAGAGTGACCACATTTC	AGGAGAGCAACCACGTAGAC
mPgc1α	GACCCCAGAGTCACCAAATGA	TTCCAGAGAGTTCCACACTTAAGG T
hXbp1	GCTGAAGAGGAGGCGGAAG	GTCCAGAATGCCCAACAGG

Table S2

Gene name	Uniprot	LOG10 p-value	LOG2 fold change	Tissue specificity	Location
Apcs	P12246	8,19	2,44	Liver	Blood
Ahsg	P29699	6,67	0,96	Liver	Blood
Serpinf2	Q61247	4,23	0,57	Liver	Blood
Apob	E9Q414	3,88	0,65	Liver	Blood
Serpina3n	Q91WP6	3,69	0,36	Liver	Blood
C5	P06684	3,65	-0,23	Liver	Blood
Serpina6	Q06770	3,48	-0,59	Liver	Blood
Ttr	P07309	3,44	-0,33	Brain, liver, retina	Blood
Fn1	P11276	3,12	0,26	Ductus deference, placenta	Blood
Gpx3	P46412	3,01	-0,43	Kidney, thyroid gland	Blood
Azgp1	Q64726	2,88	-0,44	Liver, salivary gland	Blood
Itih4	A6X935	2,67	0,38	Liver	Blood
Serpina3m	Q03734	2,65	0,33	Liver	Blood
Gsn	P13020	2,63	-0,35	Not specific	Blood
Mbl1	P39039	2,52	-0,42	Liver	Blood
Cd5l	Q9QWK4	2,48	-0,92	Lymphoid tissues	Blood
F5	O88783	2,39	0,79	Brain, liver, placenta	Blood
Saa4	P31532	2,28	-0,34	Liver	Blood
Hrg	Q9ESB3	2,21	-0,32	Liver	Blood
Fcn1	O70165	2,15	-0,48	Blood, bone marrow	Blood
Jchain	P01592	2,11	-0,69	Lymphoid tissues	Blood
Ig kappa chain V-VI	P01679	2,10	-0,68	Lymphoid tissues	Blood
Pglyrp2	Q8VCS0	2,05	-0,37	Liver	Blood
Ighm	P01872	1,97	-0,90	Lymphoid tissues	Blood
Actg1	P63260	1,93	-0,38	Not specific	Intracellular
Me1	P06801	1,80	-1,15	Not specific	Intracellular
Serpina1b	P22599	1,74	0,40	Liver	Blood
Ig heavy chain V region	P06330	1,72	-0,66	Lymphoid tissues	Blood
Adipoq	Q60994	1,64	-0,51	Adipose tissue, breast	Blood
Ig kappa chain V-V	P01645	1,51	-0,74	Lymphoid tissues	Blood
Ig heavy chain V-III	P01799	1,48	-0,73	Lymphoid tissues	Blood
Igkc	P01837	1,37	-0,61	Lymphoid tissues	Blood
Ig kappa chain V-III	P01656	1,31	-1,07	Lymphoid tissues	Blood
